# NBR1 is a critical step in the repression of thermogenesis of p62-deficient adipocytes through PPARγ

**DOI:** 10.1038/s41467-021-23085-0

**Published:** 2021-05-17

**Authors:** Jianfeng Huang, Juan F. Linares, Angeles Duran, Wenmin Xia, Alan R. Saltiel, Timo D. Müller, Maria T. Diaz-Meco, Jorge Moscat

**Affiliations:** 1 Sanford Burnham Prebys Medical Discovery Institute, La Jolla, CA USA; 2 Department of Pathology and Laboratory Medicine, Weill Cornell Medicine, New York, NY USA; 3 Sandra and Edward Meyer Cancer Center, Weill Cornell Medicine, New York, NY USA; 4 Division of Metabolism and Endocrinology, Department of Medicine, University of California, San Diego, La Jolla, CA USA; 5 Institute for Diabetes and Obesity, Helmholtz Diabetes Center at Helmholtz Centre Munich, Oberschleißheim, Germany; 6 German Center for Diabetes Research (DZD), Neuherberg, Germany; 7 Department of Pharmacology and Experimental Therapy, Institute of Experimental and Clinical Pharmacology and Toxicology, Eberhard Karls University Hospitals and Clinics, Tübingen, Germany

**Keywords:** Nuclear receptors, Fat metabolism

## Abstract

Activation of non-shivering thermogenesis is considered a promising approach to lower body weight in obesity. p62 deficiency in adipocytes reduces systemic energy expenditure but its role in sustaining mitochondrial function and thermogenesis remains unresolved. NBR1 shares a remarkable structural similarity with p62 and can interact with p62 through their respective PB1 domains. However, the physiological relevance of NBR1 in metabolism, as compared to that of p62, was not clear. Here we show that whole-body and adipocyte-specific ablation of NBR1 reverts the obesity phenotype induced by p62 deficiency by restoring global energy expenditure and thermogenesis in brown adipose tissue. Impaired adrenergic-induced browning of p62-deficient adipocytes is rescued by NBR1 inactivation, unveiling a negative role of NBR1 in thermogenesis under conditions of p62 loss. We demonstrate that upon p62 inactivation, NBR1 represses the activity of PPARγ, establishing an unexplored p62/NBR1-mediated paradigm in adipocyte thermogenesis that is critical for the control of obesity.

## Abstract (web summary)

p62 is a signaling hub protein that contributes to the control of energy expenditure. Here the authors investigate the relative roles of p62 and a similar protein, NBR1, and show that NBR1 is required for the repression of thermogenesis in adipocytes occurring in the absence of p62.

## Introduction

There are at least three main morphologically and functionally different adipocyte types: white, brown, and beige. Unlike white adipocytes, which are specialized in the storage of chemical energy in the form of triglycerides, classical brown adipocytes (BAs) are found in the interscapular area (termed interscapular brown adipose tissue, iBAT) in rodents and generate heat during cold exposure by an adaptive mechanism called non-shivering thermogenesis^1^. This process requires the expression of the uncoupling protein 1 (UCP1) in the inner mitochondrial membrane to uncouple oxidative phosphorylation from ATP regeneration, thereby dissipating the energy from electron transport as heat^2^. Active BAT is detected in cervical, supraclavicular, paravertebral, and deep neck regions, and is acutely induced by cold exposure. Since the amount of metabolic active BAT inversely correlates with body mass index in adult humans^3–5^, and BAT is believed to help reduce adiposity, the epidemic of obesity and diabetes has greatly increased the interest in this type of metabolically active type of fat^6^. A better understanding of BAT biology and physiology would greatly help in the identification of effective therapeutic targets for obesity and related metabolic diseases^7^.

p62 (encoded by *Sqstm1*) has been proposed as an autophagy adaptor required for the packing and delivery of polyubiquitinated, misfolded proteins, and dysfunctional organelles for their clearance through autophagy during basal detoxification or waste removal in response to metabolic stress^8^. However, it is also well recognized that the function of p62 extends beyond autophagy^9–12^. p62 acts as a multifunctional signaling hub due to its ability to interact with different key signaling proteins through well-defined structural elements^8^. Such interactions account for p62’s roles in pathways controlling inflammation, cell death, survival, and metabolism^9,13^. With respect to metabolism, our previous results showed that total body inactivation of p62 resulted in mature-onset obesity due to reduced energy expenditure (EE)^14,15^. We also demonstrated that the selective inactivation of p62 in adipocytes, and also specifically in BAT, recapitulated the impaired EE and the obesity phenotype of total body knockout (KO) mice^16–18^. Therefore, adipocyte’s p62 emerges as a critical regulator of energy balance and adiposity in vivo. NBR1 can also function as an autophagic adaptor with remarkable similarity in domain organization to p62, and has been suggested to form dimers with p62 through their respective PB1 domains^13,19^. We have previously reported that NBR1 inactivation in the mouse myeloid compartment impairs adipose tissue inflammation driven by M1 polarized macrophages, which results in improved glucose tolerance in obese mice^20^. Although these results demonstrated a role of NBR1 in the control of the consequences of increased adiposity, they did not address the potential intrinsic role of NBR1 in adipocyte biology.

In this work, we provide evidence supporting the concept that the nuclear interaction of p62 and NBR1 and its fine tuning of the activity of peroxisome proliferator-activated receptor γ (PPARγ) in adipocytes is essential for the stimulation of the thermogenic program of BAT and activation of adaptive thermogenesis.

## Results

### Loss of *NBR1* inhibits increased adiposity of *Sqstm1*-deficient mice

**Fig. 1:**
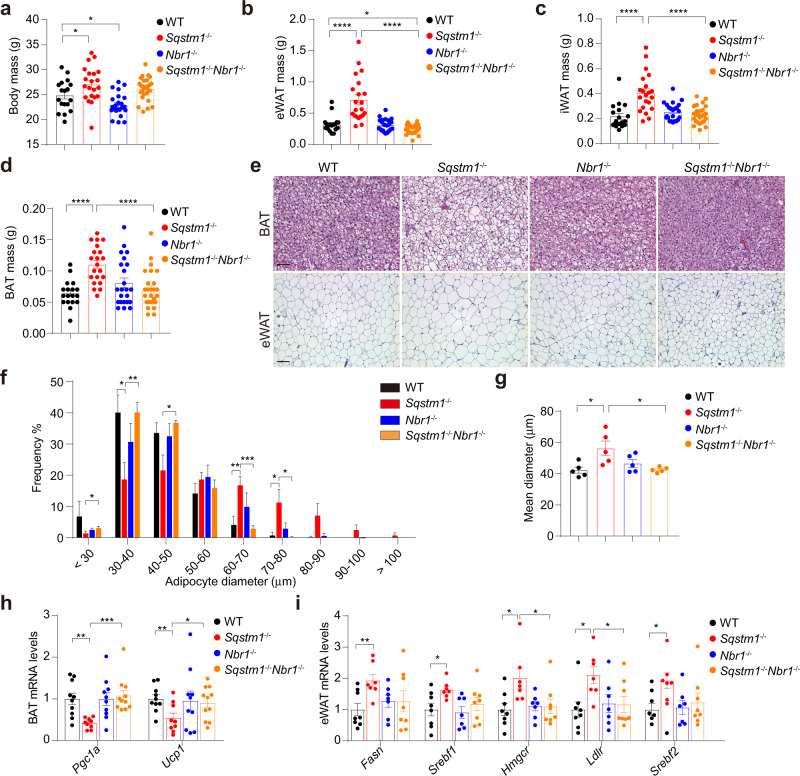
Loss of *NBR1* inhibits increased adiposity of *Sqstm1*-deficient mice. **a**–**d** Body mass (**a**) and fat tissue masses of eWAT (**b**), iWAT (**c**), and BAT (**d**) from WT and total body knockout mice at 10–12 weeks of age. WT (*n* = 18), *Sqstm1*^*–/–*^ (*n* = 21), *Nbr1*^*–/–*^ (*n* = 23), and *Sqstm1*^*–/–*^*Nbr1*^*–/–*^ (*n* = 26). *p* = 0.0422 WT vs *Sqstm1*^*–/–*^, *p* = 0.0281 vs *Nbr1*^*–/–*^ (**a**), *p* < 0.0001 WT vs *Sqstm1*^*–/–*^, *p* = 0.0142 WT vs *Sqstm1*^*–/–*^*Nbr1*^*–/–*^, *p* < 0.0001 *Sqstm1*^*–/–*^ vs *Sqstm1*^*–/–*^*Nbr1*^–/–^ (**b**), *p* < 0.0001 WT vs *Sqstm1*^*–/–*^ and *Sqstm1*^*–/–*^ vs *Sqstm1*^*–/–*^*Nbr1*^*–/–*^ (**c**, **d**). **e** Representative H&E staining of BAT and eWAT (*n* = 5, per genotype). Scale bar: 100 μm (BAT) and 200 μm (eWAT). **f**, **g** Adipocyte size measurement from H&E staining of eWAT described above (*n* = 5 mice, per genotype). Distribution range and frequency (**f**) and mean diameter of adipocyte size (**g**) were shown. *p* = 0.045 *Sqstm1*^*–/–*^ vs *Sqstm1*^*–/–*^*Nbr1*^*–/–*^ (<30), *p* = 0.0214 *Sqstm1*^*–/–*^ vs WT, *p* = 0.0071 vs *Sqstm1*^*–/–*^*Nbr1*^*–/–*^ (30–40), *p* = 0.0136 *Sqstm1*^*–/–*^ vs *Sqstm1*^*–/–*^*Nbr1*^*–/–*^ (40–50), *p* = 0.0083 *Sqstm1*^*–/–*^ vs WT, *p* = 0.0008 vs *Sqstm1*^*–/–*^*Nbr1*^*–/–*^ (60–70), *p* = 0.0323 *Sqstm1*^*–/–*^ vs WT, *p* = 0.0237 vs *Sqstm1*^*–/–*^*Nbr1*^*–/–*^ (70–80) (**f**), *p* = 0.0243 *Sqstm1*^*–/–*^ vs WT, *p* = 0.0191 vs *Sqstm1*^*–/–*^*Nbr1*^*–/–*^ (**g**). **h** qPCR analysis of thermogenesis genes in BAT. WT (*n* = 10), *Sqstm1*^*–/–*^ (*n* = 9), *Nbr1*^*–/–*^ (*n* = 10), and *Sqstm1*^*–/–*^*Nbr1*^*–/–*^ (*n* = 11). *p* = 0.0011 *Sqstm1*^*–/–*^ vs WT, *p* = 0.0003 vs *Sqstm1*^*–/–*^*Nbr1*^*–/–*^ (*Pgc1α*), *p* = 0.0073 *Sqstm1*^*–/–*^ vs WT, *p* = 0.0461 vs *Sqstm1*^*–/–*^*Nbr1*^*–/–*^ (*Ucp1*). **i** qPCR analysis of lipogenesis-related genes in eWAT. WT (*n* = 8), *Sqstm1*^*–/–*^ (*n* = 7), *Nbr1*^*–/–*^ (*n* = 7), and *Sqstm1*^*–/–*^*Nbr1*^*–/–*^ (*n* = 8). *p* = 0.0066 *Sqstm1*^*–/–*^ vs WT (*Fasn*), *p* = 0.0183 *Sqstm1*^*–/–*^ vs WT (*Srebf1*), *p* = 0.016 *Sqstm1*^*–/–*^ vs WT, *p* = 0.0364 vs *Sqstm1*^*–/–*^*Nbr1*^*–/–*^ (*Hmgcr*), *p* = 0.0105 *Sqstm1*^*–/–*^ vs WT, *p* = 0.0291 vs *Sqstm1*^*–/–*^*Nbr1*^*–/–*^ (*Ldlr*), *p* = 0.0115 *Sqstm1*^*–/–*^ vs WT (*Srebf2*). Data are presented as mean ± SEM (**a**–**d**, **f**–**i**). *p < 0.05, **p < 0.01, ***p < 0.001, ****p < 0.0001. Unpaired two-tailed Student’s *T*-test. Source data are provided as a Source Data file.

To address the role of NBR1 in adipocyte biology, we generated a total body NBR1 KO (*Nbr1*^*–/–*^) mouse line and compared its phenotype with that of total body p62 KO (*Sqstm1*^*–/–*^). Interestingly, and as previously reported^14^, *Sqstm1*^*–/–*^ mice had more body weight than wild-type (WT) mice (Fig. 1a), which was associated with increased masses of both white adipose tissue (WAT) and BAT (Fig. 1b–d). In contrast, *Nbr1*^*–/–*^ mice displayed no such metabolic phenotype (Fig. 1a–d), suggesting that NBR1 does not play a relevant role in whole-body metabolism. However, the analysis of mice with total body KO of both p62 and NBR1 (*Sqstm1*^*–/–*^*Nbr1*^*–/–*^) revealed that the increased fat weight of *Sqstm1*^*–/–*^ mice was restored to WT conditions in the absence of NBR1 (Fig. 1b–d). Consistently, while p62-deficient mice exhibited increased lipid accumulation in BAT and enlarged adipocyte size in epididymal WAT (eWAT), this phenotype was completely rescued in *Sqstm1*^*–/–*^*Nbr1*^*–/–*^ mice (Fig. 1e–g). Moreover, the mRNA levels of two master thermogenic regulators (*Ucp1* and *Pgc1α*), which were reduced in *Sqstm1*^*–/–*^ BAT, were rescued in *Sqstm1*^*–/–*^*Nbr1*^*–/–*^ BAT (Fig. 1h). Furthermore, the expression of key lipogenic genes that were increased in the eWAT of *Sqstm1*^*–/–*^ mice was partially reduced to WT levels in *Sqstm1*^*–/–*^*Nbr1*^*–/–*^ mice (Fig. 1i), likely reflecting the BAT-driven metabolic improvement in the host. These results demonstrate that NBR1 is an obligate step in the obesity phenotype unleashed by p62 deficiency, likely through the repression of the adipocyte’s thermogenic program.

### Adipocyte’s NBR1 is required for increased adiposity driven by p62 deficiency

**Fig. 2:**
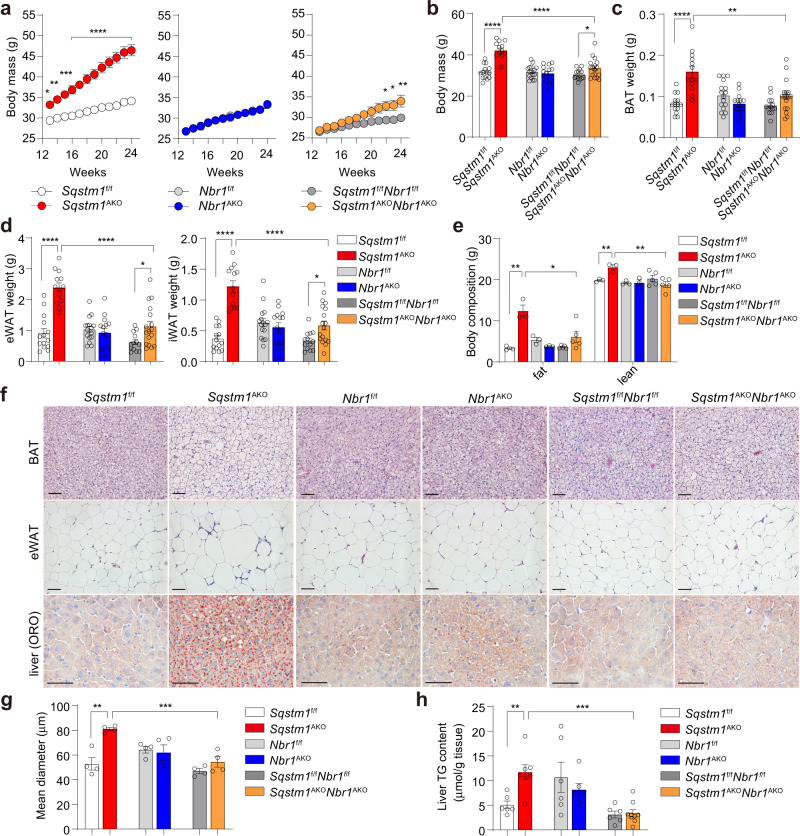
Role of adipocyte *NBR1* in the control of adiposity. **a** Recording body weight of adipocyte-specific knockout male mice and WT controls from 13 to 24 weeks of age. *Sqstm1*^f/f^ (*n* = 20), *Sqstm1*^AKO^ (*n* = 20), *Nbr1*^f/f^ (*n* = 19), *Nbr1*^AKO^ (*n* = 16), *Sqstm1*^f/f^*Nbr1*^f/f^ (*n* = 18), and *Sqstm1*^AKO^*Nbr1*^AKO^ (*n* = 15). *p* = 0.0261 (13 w), *p* = 0.0041 (14 w), *p* = 0.0003 (15 w), *p* < 0.0001 (16–24 w) *Sqstm1*^AKO^ vs *Sqstm1*^f/f^, *p* = 0.0177 (22 w), *p* = 0.012 (23 w), *p* = 0.0018 (24 w) *Sqstm1*^AKO^*Nbr1*^AKO^ vs *Sqstm1*^f/f^*Nbr1*^f/f^. **b**–**d** Body mass (**b**) and fat tissue weight of BAT (**c**) and eWAT, iWAT (**d**) from adipocyte-specific knockout male mice at 25–28 weeks of age. *Sqstm1*^f/f^ (*n* = 14), *Sqstm1*^AKO^ (*n* = 13), *Nbr1*^f/f^ (*n* = 16), *Nbr1*^AKO^ (*n* = 14). *Sqstm1*^f/f^*Nbr1*^f/f^ (*n* = 13) and *Sqstm1*^AKO^*Nbr1*^AKO^ (*n* = 17). *p* = 4.73 × 10^−7^ *Sqstm1*^AKO^ vs *Sqstm1*^f/f^, *p* = 3.08 × 10^−5^ vs *Sqstm1*^AKO^*Nbr1*^AKO^, *p* = 0.046 *Sqstm1*^AKO^*Nbr1*^AKO^ vs *Sqstm1*^f/f^*Nbr1*^f/f^ (**b**), *p* = 1.81 × 10^−5^ *Sqstm1*^AKO^ vs *Sqstm1*^f/f^, *p* = 0.001 vs *Sqstm1*^AKO^*Nbr1*^AKO^ (**c**), *p* = 2.4 × 10^−7^ *Sqstm1*^AKO^ vs *Sqstm1*^f/f^, *p* = 4.33 × 10^−6^ vs *Sqstm1*^AKO^*Nbr1*^AKO^, *p* = 0.014 *Sqstm1*^AKO^*Nbr1*^AKO^ vs *Sqstm1*^f/f^*Nbr1*^f/f^ (**d**, left), *p* = 1.48 × 10^−8^ *Sqstm1*^AKO^ vs *Sqstm1*^f/f^, *p* = 6.39 × 10^−6^ vs *Sqstm1*^AKO^*Nbr1*^AKO^, *p* = 0.01 *Sqstm1*^AKO^*Nbr1*^AKO^ vs *Sqstm1*^f/f^*Nbr1*^f/f^ (**d**, right).) **e** Fat and lean mass of 25-week-old mice of indicated genotypes by DEXA analysis. *Sqstm1*^f/f^ (*n* = 3), *Sqstm1*^AKO^ (*n* = 3), *Nbr1*^f/f^ (*n* = 3), *Nbr1*^AKO^ (*n* = 3), *Sqstm1*^f/f^*Nbr1*^f/f^ (*n* = 5), and *Sqstm1*^AKO^*Nbr1*^AKO^ (*n* = 5). *p* = 0.0034 *Sqstm1*^AKO^ vs *Sqstm1*^f/f^, *p* = 0.0237 vs *Sqstm1*^AKO^*Nbr1*^AKO^ (fat), *p* = 0.0063 *Sqstm1*^AKO^ vs *Sqstm1*^f/f^, *p* = 0.0061 vs *Sqstm1*^AKO^*Nbr1*^AKO^ (lean). **f** Representative H&E staining in BAT and eWAT and Oil Red O (ORO) staining in livers. H&E staining (*n* = 3 for *Sqstm1*^f/f^, *Sqstm1*^AKO^, *Nbr1*^f/f^ and *Nbr1*^AKO^ and *n* = 4 for *Sqstm1*^f/f^*Nbr1*^f/f^ and *Sqstm1*^AKO^*Nbr1*^AKO^), ORO staining (*n* = 3, per genotypes). Scale bar = 100 μm. **g** Adipocyte size measurement from H&E staining of eWAT described above (*n* = 4, per genoty*p*e). *p* = 0.0016 *Sqstm1*^AKO^ vs *Sqstm1*^f/f^, *p* = 0.00099 vs *Sqstm1*^AKO^*Nbr1*^AKO^. **h** TG content measurement in livers. *Sqstm1*^f/f^ (*n* = 6), *Sqstm1*^AKO^ (*n* = 7), *Nbr1*^f/f^ (*n* = 6), *Nbr1*^AKO^ (*n* = 6), *Sqstm1*^f/f^ *Nbr1*^f/f^ (*n* = 6), and *Sqstm1*^AKO^*Nbr1*^AKO^ (*n* = 9). *p* = 0.0038 *Sqstm1*^AKO^ vs *Sqstm1*^f/f^, *p* = 0.0001 vs *Sqstm1*^AKO^*Nbr1*^AKO^. Data are presented as mean ± SEM (**a**–**e**, **g**, **h**). *p < 0.05, **p < 0.01, ***p < 0.001, ****p < 0.0001. Two-way ANOVA followed by Bonferroni’s post-test (**a**). Two-tailed Student’s *T*-test (**b**–**h**). Source data are provided as a Source Data file.

To determine whether the effect of inactivating global NBR1 on obesity could be accounted for by its potential role in adipocytes, we next generated a mouse line with the adipocyte-specific deletion of NBR1 either in WT mice (*Nbr1*^AKO^) or in mice in which p62 has been selectively inactivated in adipocytes both in WAT and BAT (*Sqstm1*^AKO^). These adipocyte-selective double KO mice (*Sqstm1*^AKO^*Nbr1*^AKO^) demonstrated that the specific loss of NBR1 in p62-deficient adipocytes rescued the body weight gain of *Sqstm1*^AKO^ mice to levels close to those of the corresponding WT controls (Fig. 2a, b). That is, BAT and WAT masses and whole-body fat composition, which were increased in *Sqstm1*^AKO^ mice, were largely normalized in *Sqstm1*^AKO^*Nbr1*^AKO^ mice (Fig. 2c–e). Notably, the normalization of body weight and fat mass in *Sqstm1*^AKO^*Nbr1*^AKO^ mice is independent of age and sex (Supplementary Fig. 1a–c). Histological analyses showed that while *Sqstm1*^AKO^ mice have robustly increased adipocyte size and lipid content in BAT and eWAT, these alterations were completely abrogated in *Sqstm1*^AKO^*Nbr1*^AKO^ mice (Fig. 2f, g and Supplementary Fig. 1d). We next determined the development of fatty liver by Oil Red O (ORO) staining and quantification of hepatic lipid content. In concordance with changes in adiposity, the fatty liver phenotype of *Sqstm1*^AKO^ mice was normalized in *Sqstm1*^AKO^*Nbr1*^AKO^ mice (Fig. 2f, h). Chronic inflammation in WAT, characterized by severe macrophage infiltration, may result in systemic insulin resistance in obese diabetic animals^21^. We found that the number of crown-like structures (a hallmark of macrophage infiltration) and the expression of macrophage marker F4/80 (encoded by *Adgre1* gene), which were markedly increased in *Sqstm1*^AKO^ mice, were normalized in *Sqstm1*^AKO^*Nbr1*^AKO^ mice (Supplementary Fig. 1e, f). These findings suggest a functional dependence on NBR1 for p62 disfunction in adipocytes as the mechanism underlying the whole-body obese phenotype of *Sqstm1*^AKO^ mice.

### NBR1 inactivation in adipocytes restores glucose intolerance and insulin resistance in *Sqstm1*^AKO^ mice

**Fig. 3:**
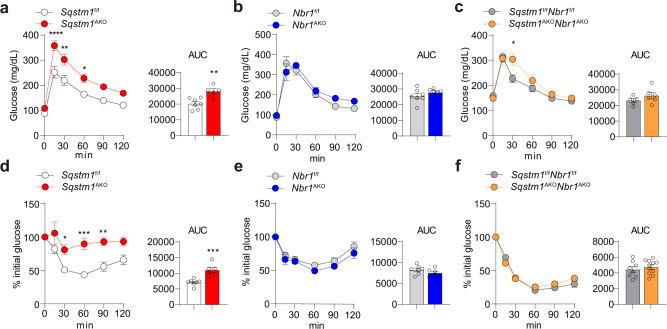
Role of adipocyte *NBR1* in the control of glucose intolerance and insulin resistance. **a**–**c** Glucose tolerance test (GTT) were performed in *Sqstm1*^AKO^ (**a**), *Nbr1*^AKO^ (**b**), *Sqstm1*^AKO^*Nbr1*^AKO^ (**c**) male mice, and their respective controls at 25–28 weeks of age. *Sqstm1*^f/f^ (*n* = 7), *Sqstm1*^AKO^ (*n* = 5), *Nbr1*^f/f^ (*n* = 7), *Nbr1*^AKO^ (*n* = 6), *Sqstm1*^f/f^*Nbr1*^f/f^ (*n* = 5), and *Sqstm1*^AKO^*Nbr1*^AKO^ (*n* = 6). Longitudinal graph: *p* < 0.0001, *p* = 0.002, *p* = 0.0319 (**a**), *p* = 0.0318 (**c**). Area under curves (AUC) were calculated from GTT. *p* = 0.0033 (**a**). **d**–**f** Insulin tolerance test (ITT) was performed in *Sqstm1*^AKO^ (**d**), *Nbr1*^AKO^ (**e**), *Sqstm1*^AKO^*Nbr1*^AKO^ (**f**) male mice and their respective controls at 25–28 weeks of age. Results are presented as percent of the initial glucose levels. *Sqstm1*^f/f^ (*n* = 7), *Sqstm1*^Ako^ (*n* = 6), *Nbr1*^f/f^ (*n* = 7), *Nbr1*^AKO^ (*n* = 6), *Sqstm1*^f/f^*Nbr1*^f/f^ (*n* = 8), and *Sqstm1*^AKO^*Nbr1*^AKO^ (*n* = 11). Longitudinal graph: *p* = 0.0273, *p* = 0.0002, *p* = 0.0035 (**d**). AUC was calculated from ITT. Data are presented as mean ± SEM (**a**–**f**). *p < 0.05, **p < 0.01, ***p < 0.001, ***p < 0.0001. Two-way ANOVA followed by Bonferroni’s post-test (**a**–**f** longitudinal graphs). Two-tailed Student’s *T*-test (**a**–**f** AUC bar graphs). Source data are provided as a Source Data file.

We next determined the impact of NBR1 deficiency in systemic glucose tolerance and insulin sensitivity. To this end, we measured glucose tolerance in mature mice of all genotypes. Interestingly, while glucose intolerance were evident characteristics of obese *Sqstm1*^AKO^ mice in GTT (Fig. 3a), this phenotype was not observed in *Nbr1*^AKO^ and *Sqstm1*^AKO^*Nbr1*^AKO^ mice (Fig. 3b, c). Next, insulin sensitivity was determined in these mice in ITT experiments. While insulin injection was insufficient to reduce the blood glucose concentrations in *Sqstm1*^AKO^ mice (Fig. 3d), *Nbr1*^AKO^ and *Sqstm1*^AKO^*Nbr1*^AKO^ mice exhibited similar responses to insulin as their flox controls (Fig. 3e, f). Although the initial glucose levels were higher in *Sqstm1*^AKO^*Nbr1*^AKO^ mice, likely due to unexpected fluctuation resulted from the short-term fasting procedure, presenting ITT data as percent of initial glucose levels clearly supports the notion that the *Sqstm1*^AKO^*Nbr1*^AKO^ mice manage to maintain insulin sensitivity but the *Sqstm1*^AKO^ mice fail to do so (Supplementary Fig. 2). Collectively, these results established that the loss of NBR1 in adipocytes protects mice from the dysfunctional glucose metabolism characteristic of adipocyte-specific p62 deficiency.

### NBR1 inactivation in adipocytes restores impaired systemic energy expenditure in *Sqstm1*^AKO^ mice

**Fig. 4:**
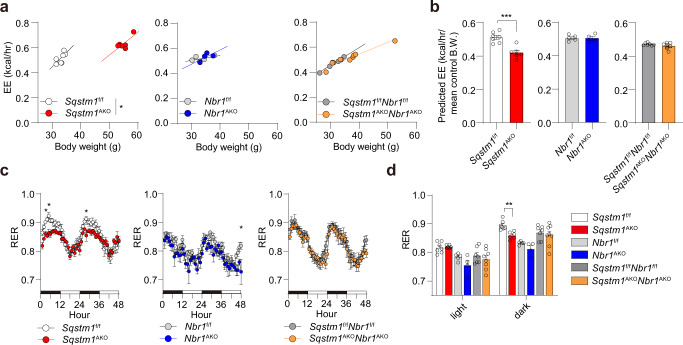
Role of *NBR1* in systemic energy expenditure. A metabolic characterization of the adipocyte-selective KO mice was performed by using an automated indirect calorimetry system (CLAMS) in male mice at 50–55 weeks of age. *Sqstm1*^f/f^ (*n* = 7), *Sqstm1*^AKO^ (*n* = 6), *Nbr1*^f/f^ (*n* = 5), *Nbr1*^AKO^ (*n* = 4), *Sqstm1*^f/f^*Nbr1*^f/f^ (*n* = 7), and *Sqstm1*^AKO^*Nbr1*^AKO^ (*n* = 8). **a** Regression plots of Energy expenditure (EE) against body weight. ANCOVA test using body weight as covariate. *p* = 0.043 *Sqstm1*^AKO^ vs *Sqstm1*^f/f^. **b** Predicted energy expenditure at the mean body weight of respective flox control mice. The mean values of body weight are 33.1 g in *Sqstm1*^f/f^, 33.46 g in *Nbr1*^f/f^, and 31.24 g in *Sqstm1*^f/f^*Nbr1*^f/f^ mice. Two-tailed Student’s *T*-test. *p* = 0.0004 *Sqstm1*^AKO^ vs *Sqstm1*^f/f^. BW body weight. **c** Respiratory exchange rate (RER) was recorded and plotted. Two-way ANOVA followed by Bonferroni’s post-test. *p* = 0.0421 (4 h), *p* = 0.0196 (5 h), *p* = 0.0308 (28 h) *Sqstm1*^AKO^ vs *Sqstm1*^f/f^, *p* = 0.0126 (48 h) *Nbr1*^AKO^ vs *Nbr1*^f/f^. **d** Quantification of respective AUC from (**c**) was analyzed by two-tailed Student’s *T*-test. *p* = 0.0012 *Sqstm1*^AKO^ vs *Sqstm1*^f/f^. Data are presented as mean ± SEM (**b**–**d**). *p < 0.05, **p < 0.01, ***p < 0.001. Source data are provided as a Source Data file.

To evaluate the role of adipocyte NBR1 in whole-body metabolic profile, especially in the context of p62 deficiency, we performed a full metabolic characterization of the adipocyte-selective KO mouse lines. In concordance with the previous study^16^, p62 ablation in adipocytes led to marked reduction in EE as determined by ANCOVA using body weight as covariate as previously described^22^ (Fig. 4a, b). Interestingly, the reduced EE of *Sqstm1*^AKO^ mice was restored in *Sqstm1*^AKO^*Nbr1*^AKO^ mice to WT levels (Fig. 4a, b). The respiratory exchange ratio (RER) denotes the preference for carbohydrates and lipids as fuels to fit the energy demand. The fact that *Sqstm1*^AKO^ mice had lower RER during the dark (feeding) phase suggests that most of the dietary carbohydrates are stored rather than being metabolized (Fig. 4c, d). Notably, this parameter was largely restored in *Sqstm1*^AKO^*Nbr1*^AKO^ mice when comparing to *Sqstm1*^AKO^ mice (Fig. 4c, d). This observation is consistent with an improvement in the whole-body metabolic rate, with no alterations in food intake and locomotor activity (Supplementary Fig. 3a, b). Furthermore, in agreement with their unaltered body weight and fat mass, *Nbr1*^AKO^ mice showed no phenotypic changes in any of the metabolic parameters investigated, including EE, RER, food intake, and locomotor activity (Fig. 4a–d and Supplementary Fig. 3a, b). These data support the notion that the loss of NBR1 in adipocytes rescues the impaired systemic EE driven by p62 deficiency.

### Adipocyte NBR1 is required for downregulation of adaptive thermogenesis in BAT and inguinal WAT of p62-deficient mice

**Fig. 5:**
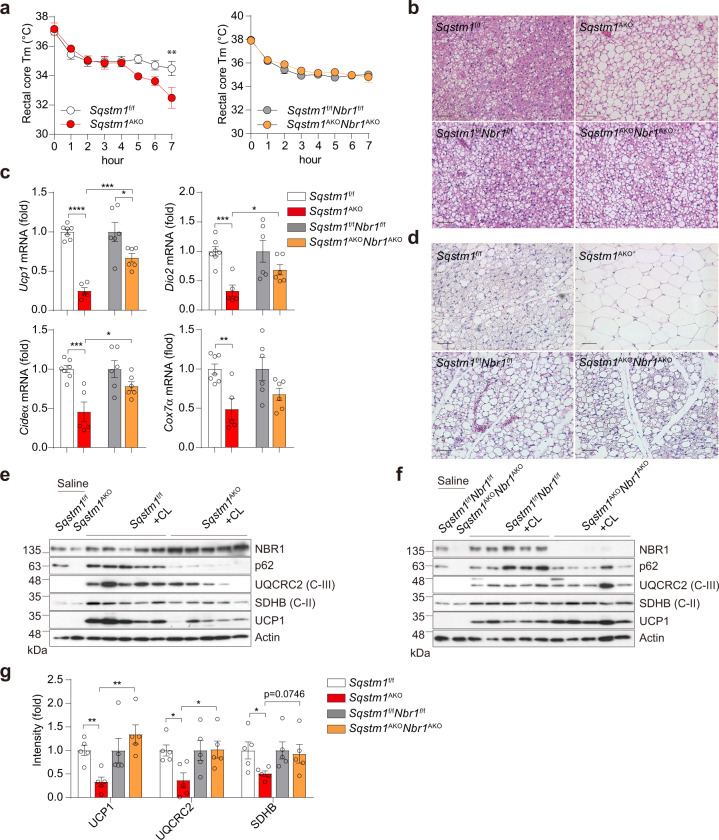
Role of NBR1 in adaptive thermogenesis in BAT and inguinal WAT. **a**–**c** Male mice at 25 weeks of age were subjected to acute cold exposure (4 °C) for 7 h to stimulate brown thermogenesis. **a** Rectal core temperature was measured for consecutive 7 h. *Sqstm1*^f/f^ (*n* = 10), *Sqstm1*^AKO^ (*n* = 6), *Sqstm1*^f/f^*Nbr1*^f/f^ (*n* = 9), and *Sqstm1*^AKO^*Nbr1*^AKO^ (*n* = 10). Two-way ANOVA followed by Bonferroni’s post-test. **b** Representative H&E staining in BAT of indicated mice (*n* = 3, per genotype). Scale bar = 100 μm. **c** qPCR analysis of thermogenesis-related genes in BAT of mice. Results are presented as change fold related to individual controls. *Sqstm1*^f/f^ (*n* = 7), *Sqstm1*^AKO^ (*n* = 5), *Sqstm1*^f/f^*Nbr1*^f/f^ (*n* = 6), and *Sqstm1*^AKO^*Nbr1*^AKO^ (*n* = 6). Two-tailed Student’s *T*-test. **d**–**g** Male mice at 25 weeks of age were injected with CL316,243 or saline as control for consecutive 5 days. **d** Representative H&E staining in iWAT of indicated mice. *Sqstm1*^f/f^ (*n* = 4), *Sqstm1*^AKO^ (*n* = 4), *Sqstm1*^f/f^*Nbr1*^f/f^ (*n* = 3), and *Sqstm1*^AKO^*Nbr1*^AKO^ (*n* = 3). Scale bar = 100 μm. **e**, **f** Immunoblot analysis of mitochondrial OXPHOS genes and UCP1 in BAT of *Sqstm1*^AKO^ (**e**) and *Sqstm1*^AKO^*Nbr1*^AKO^ (**f**) and their respective controls (*n* = 5, per genotype). **g** Densitometric quantification of gene intensity from western blot (**e**, **f**). Results are presented as change fold related to individual controls. Two-tailed Student’s *T*-test. Data are presented as mean ± SEM (**a**, **c**, **g**). *p < 0.05, **p < 0.01, ***p < 0.001, ****p < 0.0001. Source data are provided as a Source Data file.

We next tested in these mice the adaptive thermogenic capacity of BAT. To this end, mice of the different genotypes were exposed to cold (4 °C) for 7 h to stimulate their thermogenic program. In contrast to the hypothermic *Sqstm1*^AKO^ mice, the *Sqstm1*^AKO^*Nbr1*^AKO^ and *Nbr1*^AKO^ mice were able to maintain their core temperature against acute cold exposure to levels similar to their respective WT controls, suggesting intact heat generation (Fig. 5a and Supplementary Fig. 4a). Interestingly, while a “whitening” histological feature was found in *Sqstm1*^AKO^ BAT upon cold exposure, indicative of insufficient lipid mobilization and metabolization, this abnormality was largely rescued in *Sqstm1*^AKO^*Nbr1*^AKO^ mice (Fig. 5b), while *Nbr1*^AKO^ mice showed no phenotype (Supplementary Fig. 4b). Consistently, the expression of thermogenic genes (*Ucp1*, *Dio2*, *Cideα*, and *Cox7α*) in response to cold-driven sympathetic stimulation was significantly decreased in *Sqstm1*^AKO^ BAT but was largely rescued in *Sqstm1*^AKO^*Nbr1*^AKO^ BAT (Fig. 5c).

Beige adipocytes are the inducible form of thermogenic fat cells that emerge within inguinal WAT in rodents in response to a variety of external stimuli, such as chronic cold exposure and cancer cachexia^17,23^. Injection of the β_3_-adrenergic agonist CL316,243 in *Sqstm1*^AKO^*Nbr1*^AKO^ and *Nbr1*^AKO^ mice rapidly switched adipocytes from the characteristic unilocular to the multilocular cell morphology, which is typical of beige/bright cells (Fig. 5d and Supplementary Fig. 4c). However, inguinal white adipocytes in *Sqstm1*^AKO^ mice remained unilocular and enlarged in cell size (Fig. 5d), indicative of the absence of beige cells. The levels of UCP1 and mitochondrial complex proteins were upregulated by CL316,243 in *Sqstm1*^AKO^*Nbr1*^AKO^ mice, as well as that in WT controls whereas such an induction was largely diminished in *Sqstm1*^AKO^ mice (Fig. 5e–g). These results establish that p62 and NBR1 impact the thermogenesis program of both brown and beige adipocytes, and likely their mitochondrial function.

**Fig. 6:**
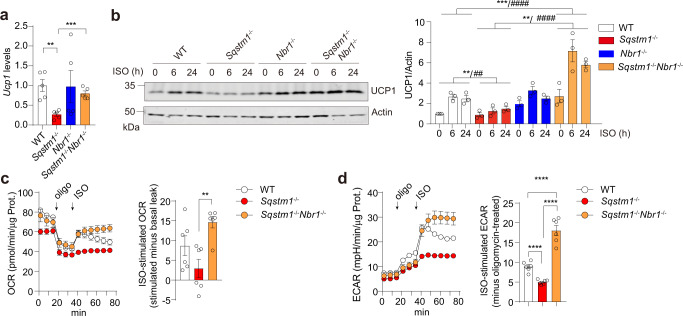
Role of NBR1 in adaptive thermogenesis in primary brown adipocytes. **a** qPCR analysis of *Ucp1* expression in primary brown adipocytes differentiated from SVF of four indicated genotypes (*n* = 5 biological replicates). **b** Immunoblot analysis of UCP1 in primary brown adipocytes of four indicated genotypes, treated with ISO (0.5 µM) for indicated time. Representative blots (left) and quantification (right) from three independent experiments were shown. **c**, **d** Immortalized SVF from WT, *Sqstm1*^*–/–*^, and *Sqstm1*^*–/–*^*Nbr1*^*–/–*^ mice were differentiated into brown adipocytes for Seahorse analyses. **c** Oxygen consumption rate (OCR) were determined (*n* = 6 biological replicates). Left: plot of OCR to time measured by Seahorse. Right: calculated ISO-stimulated respiration levels after subtracting levels of basal proton leak. **d** Extracellular acidification rate (ECAR) were measured (*n* = 6 biological replicates). Left: plot of ECAR to time measured by Seahorse. Right: calculated ISO-stimulated ECAR values after subtracting oligomycin-treated levels. Data are presented as mean ± SEM (**a**–**d**). *p < 0.05, **/^##^*p* < 0.01, ***p < 0.001, ****/^####^*p* < 0.0001. Two-tailed Student’s *T*-test (**a**, **c**, **d** bar graphs). Two-way ANOVA (**b**). Source data are provided as a Source Data file.

In this regard, our previously published results demonstrated that the loss of p62 in BAs resulted in impaired mitochondrial oxygen consumption rate (OCR)^16^. To determine the impact of NBR1 deficiency in this process, we first established primary BAs through differentiation of BAT stromal vascular fractions (SVF) isolated from either single or double KO mice. Under basal condition, *Ucp1* mRNA levels were significantly decreased in primary *Sqstm1*^*–/–*^ BAs, but that reduction was abrogated in *Sqstm1*^*–/–*^*Nbr1*^*–/–*^ BAs (Fig. 6a). While increased UCP1 protein expression was induced by the adrenergic agonist isoproterenol (ISO) in all the four genotypes, the induction was comparably lower in primary *Sqstm1*^*–/–*^ BAs than that in WT and *Sqstm1*^*–/–*^*Nbr1*^*–/–*^ BAs (Fig. 6b). We also generated immortalized brown adipocytes (iBAs). Interestingly, while the oligomycin-resistant mitochondrial OCR (basal uncoupling respiration) was decreased in *Sqstm1*^*–/–*^ iBAs, it remained unaltered in *Sqstm1*^*–/–*^*Nbr1*^*–/–*^ iBAs (Fig. 6c). Subsequent ISO injection elevated respiration levels (ISO-stimulated uncoupling OCR) in *Sqstm1*^*–/–*^*Nbr1*^*–/–*^ iBAs significantly higher than that in *Sqstm1*^*–/–*^ iBAs (Fig. 6c). BAT takes up large amounts of glucose during cold exposure in mice and humans. The cold-induced expression of glycolytic enzymes could be mimicked in vitro by β-adrenergic stimulation^24^. We found that upon ISO stimulation, *Sqstm1*^*–/–*^*Nbr1*^*–/–*^ iBAs exhibited higher ECAR relative to *Sqstm1*^*–/–*^ iBAs (Fig. 6d). These results demonstrated that the loss of NBR1 in BAs largely rescued the thermogenic and mitochondrial defects in *Sqstm1*^*–/–*^ BAT.

### p62 and NBR1 interact with PPARγ in the nucleus of brown adipocytes

**Fig. 7:**
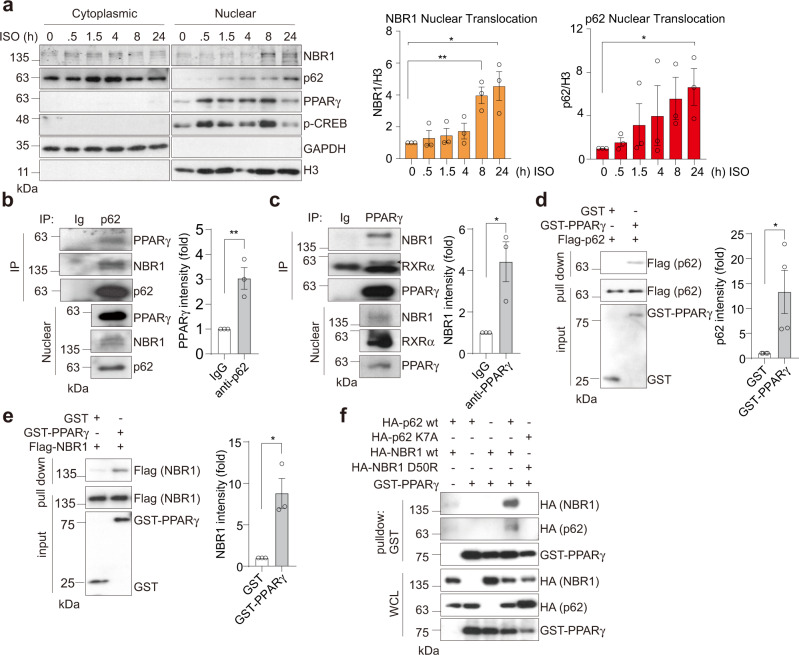
p62 and NBR1 interact with PPARγ in brown adipocytes. **a** Representative immunoblotting of p62 and NBR1 levels in cytoplasmic/nuclear fractions in primary brown adipocytes. Cells were treated with 0.5 µM isoproterenol (ISO) for indicated time. Densitometric quantification of nuclear NBR1 and p62 levels was also shown (*n* = 3 independent experiments). **b**, **c** Endogenous interaction of PPARγ with p62 and NBR1. p62 (**b**) or PPARγ (**c**) immunoprecipitates from nuclear lysates extracted from ISO and rosiglitazone-treated brown adipocytes were analyzed for the levels of specified proteins. Representative blots and densitometric quantification were shown (*n* = 3 independent experiments for both). **d**, **e** Recombinant FLAG-p62 (**d**) or FLAG-NBR1 (**e**) was incubated with GST and GST-PPARγ proteins separately and the interactions were analyzed by immunoblotting in glutathione-beads pull-down. Representative blots and densitometric quantification were shown (*n* = 4 independent experiments for **d** and *n* = 3 independent experiments for **e**). **f** HEK293T cells were transfected with cDNA vectors expressing WT/mutants of HA-p62 or HA-NBR1, and GST-PPARγ. The interacting proteins were pulled down using glutathione-beads against GST-PPARγ and analyzed by immunoblotting. Representative blots from three independent replicates with similar results were shown. Data are presented as mean ± SEM (**a**–**e**). *p < 0.05, **p < 0.01. Two-tailed Student’s *T*-test (**a**–**e**). Source data are provided as a Source Data file.

In search of the molecular crosstalk between p62 and NBR1 that might underly their role in adipocyte biology, we initially found that NBR1 expression was dramatically upregulated during brown adipogenesis, following a pattern similar to that of PPARγ expression (Supplementary Fig. 5a). Unlike NBR1, p62 showed just a marginal increase, detectable only in fully differentiated mature BAs (Supplementary Fig. 5a). Treatment with ISO that transcriptionally upregulates UCP1 and drives thermogenesis robustly induced the nuclear amounts of PPARγ and CREB, two master thermogenic regulators, but also triggered the nuclear translocation of p62 and NBR1 (Fig. 7a). We hypothesized that the impaired thermogenic and mitochondrial activity characteristic of p62 deficiency are due to a defective activation of the PPARγ transcriptional program. Since adrenergic stimulation augments the nuclear levels of p62, NBR1, and PPARγ (Fig. 7a), we speculated that they could be part of a multi-component protein complex. Consistent with this hypothesis, PPARγ and NBR1 were co-immunoprecipitated with p62 in iBAs treated with ISO and rosiglitazone (Fig. 7b and Supplementary Fig. 5b). These observations demonstrate that both p62 and NBR1 can form a complex with PPARγ. Because similar results were obtained when these precipitations were performed with purified recombinant proteins, we concluded that these interactions were direct (Fig. 7d, e). Furthermore, we found in co-transfection experiments that p62 and NBR1 influence each other’s interaction with PPARγ (Supplementary Fig. 5d). Consistently, mutations of p62 (K7A) and NBR1 (D50R) within their respective PB1 domains that abolished p62-NBR1 interaction^11,19,20^ also clearly abrogated the synergistic binding of p62 and NBR1 to PPARγ (Fig. 7f and Supplementary Fig. 5e). These results demonstrate a nuclear interaction among p62, NBR1, and PPARγ, which might influence PPARγ’s activities in BAs.

### p62 and NBR1 regulate PPARγ-RXRα heterodimerization to control thermogenesis in brown adipocytes

**Fig. 8:**
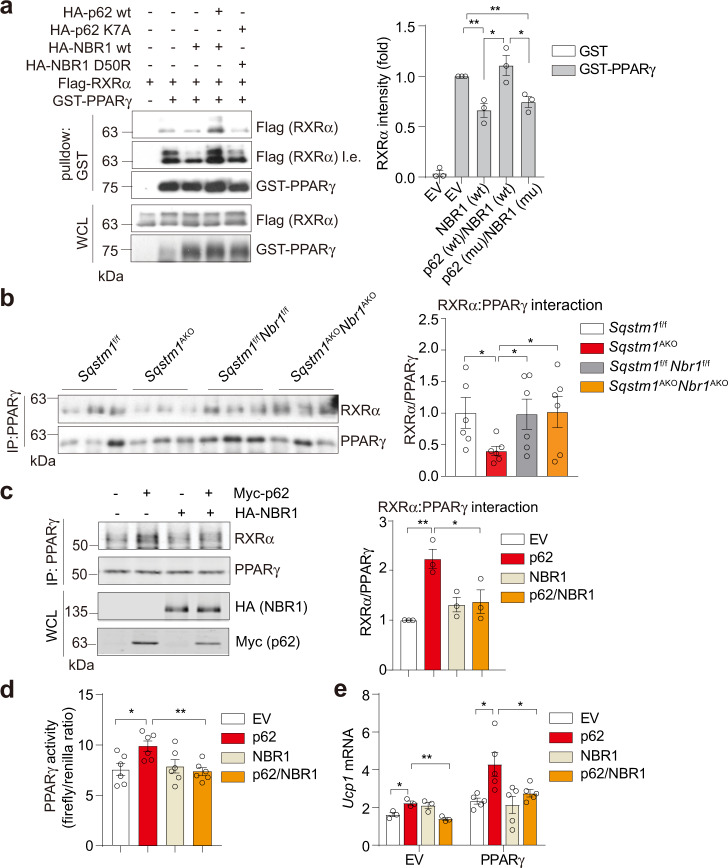
p62 and NBR1 regulate PPARγ-RXRα heterodimerization to control thermogenesis in brown adipocytes. **a** FLAG-RXRα, GST-PPARγ, WT/mutants of HA-p62, and HA-NBR1 were overexpressed in HEK293T cells and the interaction of RXRα with PPARγ was analyzed by immunoblotting in pull-downs using glutathione-beads against GST-PPARγ, in the present of NBR1 and/or p62. Representative immunoblotting and densitometric quantification were shown (*n* = 3 independent experiments). EV empty vector, wt wild-type, mu mutant. **b** Endogenous interaction of PPARγ with RXRα in BAT of mice exposed to cold for 7 h. PPARγ immunoprecipitates were analyzed by immunoblotting. Densitometric quantification was shown (*n* = 6, per *Sqstm1*^f/f^, *Sqstm1*^AKO^, *Sqstm1*^f/f^*Nbr1*^f/f^, and *Sqstm1*^AKO^*Nbr1*^AKO^). **c** Endogenous interaction of PPARγ with RXRα in double KO iBAs (sg*Sqstm1*sg*Nbr1*) reconstituting p62, NBR1, or both. PPARγ immunoprecipitates were analyzed by immunoblotting. Densitometric quantification was shown (*n* = 3 independent experiments). **d** Luciferase assay determining transcriptional activity of PPARγ in iBAs transfected with indicated cDNA vectors, cells were treated with ISO (1 µM) and rosiglitazone (1 µM) for 48 h (*n* = 6 biological replicates). **e** qPCR analysis of *Ucp1* expression in *Sqstm1*^*–/–*^*Nbr1*^*–/–*^ iBAs reconstituting p62 or NBR1 or both with/without overexpression of PPARγ. Cells were treated with ISO (1 µM) for 48 h. EV (*n* = 3 biological replicates), PPARγ (*n* = 5 biological replicates). Data are presented as mean ± SEM (**a**–**e**). *p < 0.05, **p < 0.01. Two-tailed Student’s *T*-test (**a**–**e**). Source data are provided as a Source Data file.

PPARγ functions as an obligate heterodimer with RXRα, which together bind to PPAR-responsive regulatory elements (PPRE) to activate the expression of target genes^25^. Our data also showed that NBR1 co-immunoprecipitated with RXRα in PPARγ immunoprecipitates (Fig. 7c and Supplementary Fig. 5c). Given the functional importance of the PPARγ-RXRα interaction, we hypothesized that p62 and NBR1 might play a critical role on its formation. Interestingly, the expression of NBR1 impaired the PPARγ-RXRα complex, which was restored by p62 expression (Fig. 8a). However, such a restoration was abolished by mutations that disrupt the interaction between p62 and NBR1 (Fig. 8a). Consistent with this model, the levels of PPARγ:RXRα heterodimer in cold-exposed BAT were reduced in *Sqstm1*^*–/–*^ mice but were normal in *Sqstm1*^*–/–*^*Nbr1*^*–/–*^ mice (Fig. 8b). We next reconstituted *Sqstm1* and *Nbr1* double KO iBAs with either p62 or NBR1 or both together and determined the PPARγ:RXRα heterodimerization. While p62 was able to enhance interaction between PPARγ and RXRα, NBR1 blunted p62 effects (Fig. 8c). Of great functional relevance, the luciferase assay using a reporter construct revealed an enhanced PPARγ transcriptional activity by p62, but this was reverted by NBR1 co-reconstitution (Fig. 8d). Furthermore, the expression of *Ucp1*, whose transcription is driven by PPARγ^26^, was increased upon p62 reconstitution, but the expression of NBR1 severely inhibited p62 effect (Fig. 8e). Our data support the role of p62 and NBR1 in the regulation of PPARγ:RXRα heterodimerization and PPARγ-mediated thermogenic program in BAs.

## Discussion

We reported previously that the obese and insulin-resistant phenotypes observed in the whole-body p62 deficiency resulted from reduced systemic EE^14^ that underscores the critical role of adipocyte’s p62 in sustaining β3-adrenergic signaling-induced mitochondrial function and thermogenesis in BAT^16^, as well as cancer-associated browning of subcutaneous WAT^17^. Importantly, the impaired thermogenesis is not secondary to the obese phenotype because impaired EE has previously been demonstrated in newborn p62 mutant pups and in young p62 mutant mice that yet do not differ in body weight or body composition^18^. Further evidence has been shown in isolated and in vitro cultured brown and inguinal white adipocytes harvested from either young lean non-obese p62 mutant mice^16,18^ or neonates from this study.

The severe adiposity and gain of fat mass characteristic of mature p62 mutant mice raises an important question whether glucose tolerance test should be assessed by dosing glucose by lean body mass but not by total body weight. Although dosing according to lean mass has been suggested by some studies especially when body composition is relatively similar, a larger body of literature argues against that because non-lean tissue mass like the white and brown fat and the brain can significantly contribute to whole-body glucose uptake^27–29^. This is particularly important in obese animals, in which the non-lean mass can make up to 50% of the body weight, and relative to the muscles, adipose glucose uptake is as high as 30% in obese mice^28^. Thus, total body weight has been adapted for glucose/insulin tolerance tests and normalization of EE data in this study.

Despite the marked phenotypes, the mechanisms whereby p62 deficiency in adipocytes impaired these processes was unclear from those previous studies. The data shown now demonstrate that whole-body and adipocyte-specific NBR1 ablation reverts the obese phenotype induced by p62 deficiency by restoring global EE and thermogenesis in BAT. We also establish here that these in vivo observations stem from a cell autonomous mechanism by which p62 antagonizes an unexpected nuclear function of NBR1 in PPARγ repression. NBR1 shares a remarkable structural similarity with p62 and can physically interact with p62 through their respective PB1 domains^13,19^. However, the physiological relevance of that interaction was not clear until now.

**Fig. 9:**
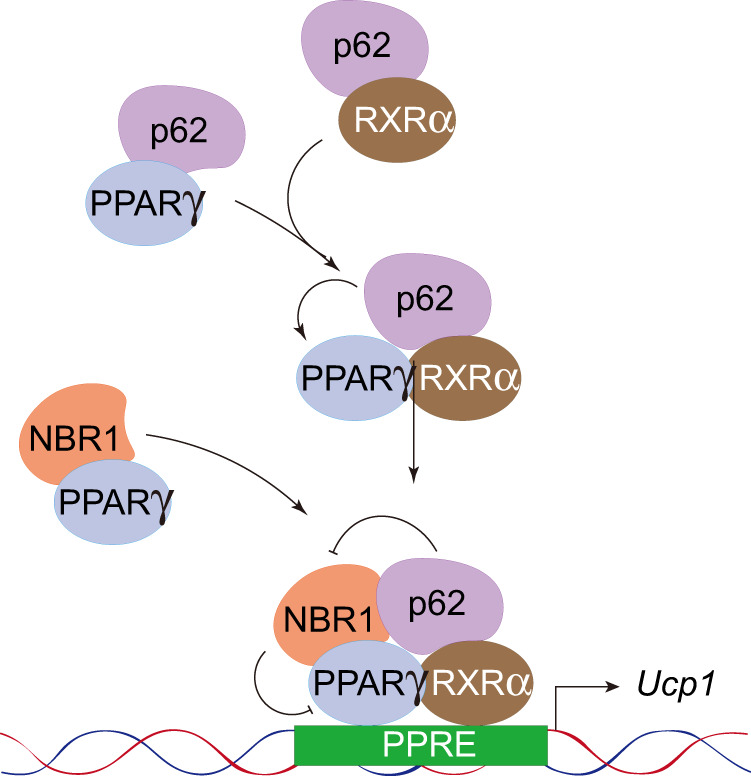
Model for the role of p62 and NBR1 in the regulation of PPARγ:RXRα heterodimerization and PPARγ-mediated thermogenic program in brown adipocytes. p62 and NBR1 translocate into the nucleus of brown adipocytes upon adrenergic stimulation and form a multi-protein complex with the nuclear receptor PPARγ and its obligate co-activator RXRα. p62 separately interacts with PPARγ and RXRα, increasing their nuclear proximity and facilitates their heterodimerization to drive thermogenic gene expression. NBR1 emerges as an opposite player that impairs PPARγ:RXRα complex formation and decreases PPARγ activity through direct interaction with PPARγ. The fact that p62 and NBR1 synergistically bind to PPARγ allows p62 to restrain NBR1 from its inhibitory binding to PPARγ. Nuclear p62 serves as a thermogenic promoter contributing to PPARγ activation in brown adipocytes.

The PPARs are members of the nuclear receptor (NR) superfamily of ligand-inducible transcription factors. PPRAγ is a critical transcriptional regulator of both WAT and BAT development as well as browning of WAT^30^. Chronic treatment with synthetic ligands of PPARγ strongly induces beige adipocyte differentiation in subcutaneous WAT^31^. PPARγ coordinates with several key co-regulators (PRDM16, PGC1α), controlling brown adipogenesis^23^. Ligand binding induces a conformational change in PPRAγ, promoting dissociation of transcriptional repressors and recruitment of co-activator, leading to activation of downstream gene expression. Collectively, our studies show that upon adrenergic stimulation, PPARγ together with p62 and NBR1 translocates into the nucleus of BAs and establish a multi-protein complex. In this way, p62 and NBR1 emerge as co-regulators of PPARγ with opposite activities. That is, whereas p62 favors PPARγ:RXRα heterodimerization to drive thermogenic gene expression, NBR1 impairs PPARγ:RXRα complex formation, decreasing PPARγ activity. Interestingly, our results are consistent with a model whereby p62 contributes to PPARγ activation by restraining NBR1 from its inhibitory binding to PPRAγ (Fig. 9). In support of this model, we show here that mutations that disrupt the p62-NBR1 interaction, or depletion of p62, allow the unleashed NBR1 to dampen PPRAγ:RXRα heterodimerization and subsequent function.

This proposed mechanism is reminiscent of the role that p62 plays in hepatic stellate cells, in which p62 facilitates the formation of a VDR:RXRα heterodimeric complex through its binding to these NRs^32^. Thus, it is conceivable that p62 could bridge different NRs, potentially increasing their proximity to respective co-regulators for an optimal transcriptional activation. The ability of p62 to interact with NRs should be considered in the context of a more general role of p62 in the nucleus. In this regard, p62 has also been shown to directly bind ATF4 and to modulate its stability in stromal fibroblasts, which is central to the ATF4-mediated metabolic reprogramming of cancer-associated fibroblasts to control tumorigenesis^33^. Direct interaction of p62 with ATF2 is required for genomic binding of ATF2 and ATF2-mediated transcription of thermogenic target genes during β-adrenergic stimulation in BAs^18^. Whether NBR1 also impacts the VDR, ATF4, and/or the ATF2 systems still needs to be addressed.

Since autophagy is suppressed by β-adrenergic signaling during fat browning^34^, it is conceivable that the accumulation of p62 and NBR1 that we describe in this paper could be the consequence of autophagy inhibition. Therefore, autophagy in this context would be a mechanism of fine tuning the signaling capabilities of p62/NBR1 in thermogenesis and adiposity. Although the role of p62 and NBR1 in mitophagy has been questioned, at least in some systems^35^, it is still possible that mitophagy inhibition might play a role in the regulation of mitochondrial levels during thermogenesis^34^, whereas the accumulated p62-NBR1 tandem described here will insure that mitochondrial biogenesis and UCP1 expression are efficiently activated transcriptionally. Therefore, the p62-NBR1 complex emerges as a central hub organizing two branches of the thermogenic program converging into mitochondrial function, acting both as autophagy adaptors and as signaling mediators through PPARγ:RXRα transcriptional signaling.

## Methods

### Animal

WT and *Sqstm1*^*–/–*^ mice were previously described^14^. aP2 cre *Sqstm1* flox (*Sqstm1*^AKO^) mice were previously described^16^. *Nbr1* flox mice were available from the previous study^20^. *Sqstm1* and *Nbr1* dual flox mice were generated by cross-bred with individual flox mice than to breed to aP2 cre to generate adipocyte-specific KO mice (*Sqstm1*^AKO^*Nbr1*^AKO^ mice). All mouse strains were generated in a C57BL/6 background. All mice were born and maintained under pathogen-free conditions. Mice were fed a normal chow diet and kept on a 12-h light/12-h dark cycle with free access to food and water in a temperature (22 ± 1 °C) and humidity (50 ± 5%) controlled room. All genotyping was done by PCR. Mice were sacrificed, and adipose tissues and liver sections were dissected. Animal handling and experimental procedures conformed to institutional guidelines and were approved by the Sanford Burnham Prebys Medical Discovery Institute Institutional Animal Care and Use Committee.

### Metabolic phenotyping

EE, O_2_ consumption rate, CO_2_ production rate, RER, food intake, and locomotor activity were assessed in male mice at 50–55 weeks of age using an automated indirect calorimetry Oxymax system of the Comprehensive Lab Animal Monitoring System (CLAMS; Promethium System) at UCSD. After 48 h of adaptation, O_2_ consumption and CO_2_ production were measured to determine the respiratory quotient and EE. EE was analyzed using ANCOVA with body weight as covariate as previously described^22,36^. Whole-body composition (fat and lean mass) was measured using Dual-Energy X-ray Absorptiometry at UCSD Animal Care Program. For glucose tolerance test, mice at 25–28 weeks of age were fasted overnight and then challenged with 1.5 g glucose per kg body weight. For insulin tolerance test, mice at 25–28 weeks of age were fasted 4 h and administrated with 0.5 U insulin per kg body weight. Glucose concentrations of tail blood were then measured by using an ACCU-CHEK Aviva (Roche) glucometer at indicated time points. For acute cold exposure, all aP2 Cre mouse lines at 25–27 weeks of age were singly housed at 4 °C in a non-bedded cage with access to water but not food for 7 h. Core body temperature was measured using a rectal probe (BAT-10, Physitemp). At the end of the experiment, BAT was resected for histological and gene expression analyses. To induce browning in WAT, mice at 25–27 weeks of age were i.p. administrated with β3-adrenergic agonist CL316,243 at 0.5 mg/kg BW for 4 days and at 1 mg/kg for the last day. After injection for 5 days, inguinal WAT was dissected for histological and gene expression analyses.

### Histological analysis

Tissues from indicated mice were isolated, rinsed in ice-cold PBS, fixed in 10% neutral buffered formalin for 24 h, dehydrated, and embedded in paraffin. Livers were embedded in Tissue Tek O.C.T. compound and snap frozen in dry ice, then kept in −80 °C. Tissue sections (5 µm) were stained with hematoxylin and eosin (H&E). Histological sections of fat pads were stained with H&E and captured under 20-fold magnification by the AxioVision LE software to determine adipocyte size. At least seven fields per section from four different mice of each genotype were randomly selected to determine the adipocyte size and number according to morphological feature using “ImageJ”-based software “Adiposoft.” Frozen liver sections (5 μm) were stained with ORO (Sigma-Aldrich) to detect lipid accumulation. Sections were fixed in paraformaldehyde and stained for 3 h in 0.5% ORO in propylene glycol, followed by 1 min incubations in 85% aqueous propylene glycol. After the slides were washed in distilled water, they were counterstained with Harris’s hematoxylin for 10 s.

### Lipid analysis

For determination of lipids mass, liver sample were washed with PBS and frozen. Total lipids were isolated from homogenates by Folch extraction. Briefly, around 50 mg tissue samples were homogenized in 1 ml methanol, homogenates were further mixed with 2 ml chloroform and rotated mildly for 2 h to extract lipid. Samples were then mixed roughly with 1 ml H_2_O for 30 s to separate phases. The lipid-containing organic phase (bottom) was collected and dried by nitrogen. Total lipids were dissolved in PBS containing 1% Triton X-100, followed by quantification by kits. The tissue lipid concentrations were determined spectrophotometrically (Wako Diagnostics, USA) and normalized to tissue weight.

### Cell culture

HEK293T/HEK293FT cells were purchased from ATCC. Primary and iBAs were generated in house. HEK293T/HEK293FT cells and iBAs were cultured in Dulbecco’s Modified Eagles Medium (DMEM, Corning) supplemented with 10% fetal bovine serum (FBS), 2 mM glutamine, and primary cells were cultured in DMEM/F-12 supplemented with GlutaMAX (Gibco) and 10% FBS. All cells were maintained in an atmosphere of 95% air and 5% CO_2_. Only cells that were tested negative for mycoplasm were used for experiments.

### Generation of primary and immortalized brown adipocytes

For preparation of primary BA, BAT was excised from neonates of WT, *Sqstm1*^*–/–*^, *Nbr1*^*–/–*^, and *Sqstm1*^*–/–*^*Nbr1*^*–/–*^ mice regardless of gender, and minced in 2 ml PBS, then added with collagenase D (1.5 U/ml), dispase II (2.4 U/ml), and CaCl_2_ (10 mM) and the tissues were incubated at 37 °C with gentle shaking (30 min). Larger particles were removed using a 100 μm cell strainer, and the filtrates were centrifuged at 500 g for 5 min three times to pellet SVF and remove collagenase residue. Isolated SVF were seeded in culture dish for 4 days to eliminate the unattached dead cell/white blood cell populations. SVF including adipogenic precursors were passed into 6-well plate for in vitro differentiation. Two days post confluence, differentiation was initiated by induction cocktail (DMEM/F-12 containing 10% FBS, 2 μg/ml dexamethasone, 0.5 mM isobutylmethylxanthine, 125 µM indomethacine, 1 nM T3, 0.5 μg/ml insulin, and 1 µM rosiglitazone) for 2 days, followed by maintenance cocktail (DMEM/F-12 containing 1 nM T3, 0.5 μg/ml insulin, and 1 µM rosiglitazone and 10% FBS) for another 5 days to become fully differentiated. To generate iBAs, BAT SVF were immortalized by retroviral pBabe-zeo-LT-ST (SV40) and selected by Zeocin. Single cell clone was selected and tested for differentiation capacity. All selected clones used in this study maintain efficient differentiation potential and express the BA marker UCP1. Immortalized SVF are cultured in DMEM supplemented with 10% FBS (without pyruvate). For differentiation of iBAs, the induction cocktail contains dexamethasone (2 µg/ml), isobutylmethylxanthine (0.5 mM), indomethacine (125 µM), rosiglitazone (0.5 µM), T3 (1 nM), and insulin (5 µg/ml) in growth medium. After 2 days, cells were maintained in growth medium supplemented only with rosiglitazone, T3, and insulin till day 7 for experiments. For transfection assay, differentiating cells at day 5 were transfected with indicated vectors using X-tremeGENE HP transfection reagent (Roche). To stimulate thermogenesis, cells were treated with ISO (Sigma-Aldrich) for indicated times when cells were fully differentiated.

### Generation of knockout cell by CRISPR/Cas9

To knockout p62 and NBR1 in immortalized SVF cells, 20-nucleotide single-guide RNA sequences targeting the mouse genes (GACUCUCCCUGCAGAGAAGA for Sqstm1 and CUACAGAUGCAAGUCCACGA for Nbr1) were purchased from Synthego and transduced into cells with recombinant *Streptococcus pyogenes* Cas9 protein (Truecut Cas9 Protein v2, Thermo) using Neon Electroporation System (Invitrogen). Single clones were expanded and screened for p62 and NBR1 expression by protein immunoblotting.

### Cytoplasmic and nuclear fractionation

Adipocytes were differentiated in P100 dishes. At day 7, mature adipocytes were lysed on ice with Buffer A (20 mM Tris-HCl at pH7.9, 1.5 mM MgCl_2_, 10 mM KCl) with phosphatase and proteinase inhibitors. Lysates were centrifuged at 750 g for 5 min at 4 °C. The supernatant was collected and centrifuged at 9000 g for 10 min (cytoplasmic fraction). The pellets from previous centrifuge were washed by Buffer A to remove cytoplasmic contamination, then resuspended in Buffer C (20 mM Tris-HCl at pH7.9, 1.5 mM MgCl_2_, 0.42 M NaCl) followed by sonication. Lysates were centrifuged at 16,800 g for 15 min and the resulting supernatant was collected as nuclear fraction.

### Immunoprecipitation

For total cell lysis, cells were rinsed once with ice-cold PBS and lysed on mice in σ3 lysis buffer (25 mM Tris-HCl at pH 8.0, 100 mM NaCl, 1% Triton X-100, 10% glycerol) with phosphatase and proteinase inhibitors. Lysates were centrifuged at 13,000 g for 15 min at 4 °C to remove cell debris. For tissue extracts, BAT was homogenized in 0.5 ml ice-cold lysis buffer (50 mM HEPES at pH7.5, 150 mM NaCl, 2.5 mM EDTA, 2.5 mM EGTA, 1% NP-40. 10% glycerol). Homogenates were centrifuged at 13,000 g for 15 min at 4 °C to remove fat layer and insoluble material. Protein content of lysates was quantified by *DC* Protein Assay Kit (Bio-Rad). For immunoprecipitation, 1 mg proteins of tissue or total cell lysates, 3–4 mg proteins of nuclear extracts from cells (for immunoprecipitating endogenous protein), or 0.5 mg of total cell lysates (for co-transfection immunoprecipitation) were pre-cleared with 30 μl 50% slurry of protein G agarose (Genesee Scientific) for 30 min. Then 2 μg of primary antibodies (anti-p62, Progen, #GP62-C; anti-PPARγ, Santa Cruz Biotechnology, #sc-7273; anti-FLAG, Sigma-Aldrich, #P2983) or control immunoglobulins were added to the lysates and incubated with rotation overnight at 4 °C. The next day, 30 μl 50% slurry of protein G was added to the extracts for additional 1 h. Immunoprecipitates were obtained by centrifuge at 2000 g at 4 °C for 5 min, followed by washing several times with BC300 buffer (50 mM Tris-HCl at pH7.9, 300 mM KCl, 2 mM EDTA, 10% glycerol, 0.1% NP-40) for nuclear extract, or the same lysis buffers for total cell lysates and tissue lysates. Immunoprecipitated proteins were denatured by adding 10 μl of sample buffer and boiled for 10 min before subjection to immunoblotting.

### GST pull-down assay

For the purification of Flag-p62 and Flag-NBR1 proteins, HEK293T cells in P150 dishes were transfected with 20 μg cDNA vectors of each genes. After 48 h, cells were lysed in lysis buffer (50 mM Tris-HCl at pH7.4, 150 mM NaCl, 1 mM EDTA, 1% Triton X-100) with phosphatase and protease inhibitors on ice for 30 min. After centrifugation at 13,000 g at 4 °C for 15 min, cell lysates were added with 20 μl slurry of Anti-FLAG M2 affinity gel (Sigma-Aldrich) for 2 h. Then, immunoprecipitates were centrifuged at 2000 g at 4 °C for 5 min and washed with TBS buffer (50 mM Tris-HCl at pH7.4, 150 mM NaCl) three times to remove unspecific binding. FLAG-tagged proteins were eluted by 3XFLAG peptide (Sigma-Aldrich) at 100 µg/ml for 30 min at 4 °C in gentle shaking. Eluted proteins were aliquoted and stored in −80 °C immediately. To generate GST, GST-fused PPARγ proteins, HEK293T cells in P100 dishes were transfected with 10 μg cDNA vectors of each genes. After 48 h, cells were lysed on ice for 30 min in RIPA lysis buffer with phosphatase and protease inhibitors. After centrifugation at 13,000 g at 4 °C for 15 min, cell lysates were added with 30 μl 50% slurry of glutathione agarose (Thermo Scientific) for 4 h followed by washing with RIPA buffer for four times. For in vitro pull-down assay, 20 μl of glutathione agarose-bound GST-PPARγ, GST were mixed with 1 μl of eluted FLAG-p62 or FLAG-NBR1 in 500 μl of NETE-N binding buffer (50 mM Tris-HCl at pH 8.0, 100 mM NaCl, 6 mM EDTA, 0.5% NP-40) with phosphatase and protease inhibitors for 1 h. After that, samples were washed several times with 1 ml of binding buffer and proteins were denatured by adding 20 μl of sample buffer followed by boiling for 10 min, subjected to immunoblotting. For the co-transfection pull-down assay, HEK293T cells in P60 dishes were transfected with 4 μg cDNA vectors of HA-tagged p62 or NBR1, FLAG-tagged RXRα, and GST-tagged PPARγ. After 48 h, cells were lysed on ice for 30 min in lysis buffer (50 mM HEPES at pH7.5, 150 mM NaCl, 2.5 mM EDTA, 2.5 mM EGTA, 1% NP-40. 10% glycerol) with phosphatase and protease inhibitors. After centrifugation at 13,000 g at 4 °C for 15 min, cell lysates were added with 30 μl 50% slurry of glutathione agarose beads overnight followed by washing with lysis buffer for four times. Proteins pulled down by GST-PPARγ were denatured by adding 40 μl of sample buffer followed by boiling for 10 min, subjected to immunoblotting.

### Immunoblot analysis

Protein extracts were separated by SDS-PAGE and transferred to Immobilon-P PVDF membranes (Millipore). After blocking with 5% nonfat dry milk in Tris-buffered saline and 0.1% Tween, the membranes were incubated overnight at 4 °C with the indicated antibodies. The following antibodies were used: GAPDH (Santa Cruz Biotech; #sc-32233; Dilution: 1:20,000), β-actin (Sigma-Aldrich; #A1978; Dilution 1:20,000), H3 (Abcam; #ab1791; Dilution 1:25,000), UCP1 (Abcam; #ab10983; Dilution: 1:6,000), alpha Tubulin (Santa Cruz Biotech; #sc-8035; Dilution: 1:1000), phospho-CREB (Ser133) (Cell signaling; #9198; Dilution 1:1,000), PPARγ (Santa Cruz Biotech; #sc-7273; Dilution 1:800), PPARγ (Cell signaling; #2443; Dilution 1:1,000), RXRα (Santa Cruz Biotech; #sc-553; Dilution 1:500), RXRα/β/γ (Santa Cruz Biotech; #sc-774; Dilution 1:500), p62 (Rodent Specific) (Cell signaling; #23214; Dilution 1:1000), p62, (Progen; #GP62-C; Dilution 1:1000), p62 (Thermo Scientific; #PA5-20839; Dilution 1:1000), NBR1 (Santa Cruz Biotech; #sc-130380; Dilution 1:800), SDHB (Santa Cruz Biotech; #sc-271548; Dilution 1:2,000), UQCRC2 (Santa Cruz Biotech; #sc-390378; Dilution 1:2,000), GST (Santa Cruz Biotech; #sc-138; Dilution 1:500), HA (Santa Cruz Biotech; #sc-7392; Dilution 1:500), FLAG (Sigma-Aldrich; #F1804; Dilution 1:4,000), Myc (Santa Cruz Biotech; #sc-40; Dilution 1:500). After 1 h incubation with the appropriate secondary horseradish peroxidase-conjugated antibodies including anti-mouse IgG1(BD Biosciences; #550331; Dilution 1:3,000) and anti-rabbit IgG (Dako; #E0432; Dilution 1:3,000), the immune complexes were detected by chemiluminescence (Thermo Scientific). The gels/blots with the same experiment were processed in parallel for optimal quantitative comparisons. Densitometric quantification of protein intensity from western blot was assessed by ImageJ software. Images of uncropped blots are provided in the Source Data file.

### Luciferase assay

PPAR transcriptional activity was monitored in vitro using a reporter construct consisting of three PPRE copies upstream of a luciferase reporter. At day 5 of differentiation, BAs differentiated from immortalized SVF were transiently transfected with the following plasmids using X-tremeGENE: PPREx3-TK-luc (Addgene#1015), pRL-TK (control Renilla), HA-p62, HA-NBR1 or Flag-PPARγ1 (Addgene#78769). The level of promoter activity was evaluated by determining the firefly luciferase activity relative to renilla luciferase activity using the Dual Luciferase Assay System (Promega) according to the manufacturer’s instruction.

### RNA analysis

Total RNA from mouse tissues and cultured cells was isolated using the TRIZOL reagent (Invitrogen) and the RNeasy Mini Kit (QIAGEN), followed by DNase treatment. After quantification using a Nanodrop 1000 spectrophotometer (Thermo Scientific), RNA was reverse transcribed using random primers and MultiScribe Reverse Transcriptase (Applied Biosystems). Gene expression was analyzed by amplifying 20 ng of the complementary DNA using the CFX96 Real Time PCR Detection System with SYBR Green Master Mix (Bio-Rad). The amplification parameters were set at 95 °C for 30 s, 58 °C for 30 s, and 72 °C for 30 s (40 cycles total). Gene expression values for each sample were normalized to the 18S RNA. A complete list of all primers used is listed in Supplementary Table 1.

### Measurement of respiration in adipocytes

The cellular OC of BAs was determined using an XFp Extracellular Flux Analyzer and analyzed by Agilent Seahorse Wave Software (Seahorse Bioscience). Prior to assay, 10,000 immortalized SVF cells were seeded into XFp microplates. One day later, Adipogenic differentiation was initiated using a protocol mentioned above. Seven days post differentiation, adipocyte culture medium was changed to XF basal medium containing 5 mM glucose, 1 mM pyruvate, and 2 mM GlutaMAX. The basal uncoupled OCR was determined using 1 μM oligomycin. To determine the impact of ISO stimulation in uncoupled OCR. Then, 5 μM ISO was injected three cycles after oligomycin injection. Oxygen consumption values were normalized to protein content.

### Statistical analysis

GraphPad Prism software (v. 8.3.0) was used for graphing and statistical analysis. For comparison between two groups, datasets were analyzed by Unpaired Student’s two-tailed *T*-test. Multiple comparisons were analyzed by two-way ANOVA to determine the statistical significance between groups on the basis of one variable. Differences in EE were calculated using ANCOVA with body weight as covariate using SPSS (version 24). Values of *p* < 0.05 were considered as significantly different.

### Reporting summary

Further information on research design is available in the Nature Research Reporting Summary linked to this article.

## Supplementary information

Supplementary Information

Reporting Summary

## Data Availability

The data that support the findings of this study are provided in the data source file and available from the corresponding author upon reasonable request. The statistical *p* value from GraphPad Prism or SPSS reports is provided in the individual figure legends. Source Data are provided with this paper.

## Source data

Source Data

## References

[1] Cannon B, Nedergaard J (2004). Brown adipose tissue: function and physiological significance. Physiol. Rev..

[2] Betz MJ, Enerback S (2018). Targeting thermogenesis in brown fat and muscle to treat obesity and metabolic disease. Nat. Rev. Endocrinol..

[3] Cypess AM (2009). Identification and importance of brown adipose tissue in adult humans. N. Engl. J. Med..

[4] van Marken Lichtenbelt WD (2009). Cold-activated brown adipose tissue in healthy men. N. Engl. J. Med..

[5] Saito M (2009). High incidence of metabolically active brown adipose tissue in healthy adult humans: effects of cold exposure and adiposity. Diabetes.

[6] Wu J (2012). Beige adipocytes are a distinct type of thermogenic fat cell in mouse and human. Cell.

[7] Harms M, Seale P (2013). Brown and beige fat: development, function and therapeutic potential. Nat. Med..

[8] Moscat J, Diaz-Meco MT (2009). p62 at the crossroads of autophagy, apoptosis, and cancer. Cell.

[9] Moscat J, Karin M, Diaz-Meco MT (2016). p62 in cancer: signaling adaptor beyond autophagy. Cell.

[10] Duran A (2008). The signaling adaptor p62 is an important NF-kappaB mediator in tumorigenesis. Cancer Cell.

[11] Duran A (2011). p62 is a key regulator of nutrient sensing in the mTORC1 pathway. Mol. Cell.

[12] Valencia T (2014). Metabolic reprogramming of stromal fibroblasts through p62-mTORC1 signaling promotes inflammation and tumorigenesis. Cancer Cell.

[13] Moscat J, Diaz-Meco MT (2011). Feedback on fat: p62-mTORC1-autophagy connections. Cell.

[14] Rodriguez A (2006). Mature-onset obesity and insulin resistance in mice deficient in the signaling adapter p62. Cell Metab..

[15] Lee SJ (2010). A functional role for the p62-ERK1 axis in the control of energy homeostasis and adipogenesis. EMBO Rep..

[16] Muller TD (2013). p62 links beta-adrenergic input to mitochondrial function and thermogenesis. J. Clin. Invest..

[17] Huang J (2018). Adipocyte p62/SQSTM1 suppresses tumorigenesis through opposite regulations of metabolism in adipose tissue and tumor. Cancer Cell.

[18] Fischer K (2020). The scaffold protein p62 regulates adaptive thermogenesis through ATF2 nuclear target activation. Nat. Commun..

[19] Moscat J, Diaz-Meco MT, Albert A, Campuzano S (2006). Cell signaling and function organized by PB1 domain interactions. Mol. Cell.

[20] Hernandez ED (2014). A macrophage NBR1-MEKK3 complex triggers JNK-mediated adipose tissue inflammation in obesity. Cell Metab..

[21] Weisberg SP (2003). Obesity is associated with macrophage accumulation in adipose tissue. J. Clin. Invest..

[22] Tschop MH (2011). A guide to analysis of mouse energy metabolism. Nat. Methods.

[23] Inagaki T, Sakai J, Kajimura S (2016). Transcriptional and epigenetic control of brown and beige adipose cell fate and function. Nat. Rev. Mol. Cell Biol..

[24] Basse AL (2017). Regulation of glycolysis in brown adipocytes by HIF-1alpha. Sci. Rep..

[25] Chawla A, Repa JJ, Evans RM, Mangelsdorf DJ (2001). Nuclear receptors and lipid physiology: opening the X-files. Science.

[26] Cao W (2004). p38 mitogen-activated protein kinase is the central regulator of cyclic AMP-dependent transcription of the brown fat uncoupling protein 1 gene. Mol. Cell Biol..

[27] Fam BC (2012). Normal muscle glucose uptake in mice deficient in muscle GLUT4. J. Endocrinol..

[28] Makarova E (2019). Decreases in circulating concentrations of long-chain acylcarnitines and free fatty acids during the glucose tolerance test represent tissue-specific insulin sensitivity. Front Endocrinol. (Lausanne).

[29] Higgins J, Proctor D, Denyer G (1999). Aging changes tissue-specific glucose metabolism in rats. Metabolism.

[30] Ahmadian M (2013). PPARgamma signaling and metabolism: the good, the bad and the future. Nat. Med..

[31] Ohno H, Shinoda K, Spiegelman BM, Kajimura S (2012). PPARgamma agonists induce a white-to-brown fat conversion through stabilization of PRDM16 protein. Cell Metab..

[32] Duran A (2016). p62/SQSTM1 by binding to vitamin D receptor inhibits hepatic stellate cell activity, fibrosis, and liver cancer. Cancer Cell.

[33] Linares JF (2017). ATF4-induced metabolic reprograming is a synthetic vulnerability of the p62-deficient tumor stroma. Cell Metab..

[34] Altshuler-Keylin S (2016). Beige adipocyte maintenance is regulated by autophagy-induced mitochondrial clearance. Cell Metab..

[35] Lazarou M (2015). The ubiquitin kinase PINK1 recruits autophagy receptors to induce mitophagy. Nature.

[36] Speakman JR, Fletcher Q, Vaanholt L (2013). The ‘39 steps’: an algorithm for performing statistical analysis of data on energy intake and expenditure. Dis. Model Mech..

