# Supplementary material for: NBR1 is a critical step in the repression of thermogenesis of p62-deficient adipocytes through PPARγ
Source: Nat Commun. 2021 May 17;12:2876. doi: 10.1038/s41467-021-23085-0 (PMC8129077; doi:10.1038/s41467-021-23085-0)
Supplement: Supplementary file 1 — Supplementary Information [file 41467_2021_23085_MOESM1_ESM.pdf]

SUPPLEMENTARY INFORMATION

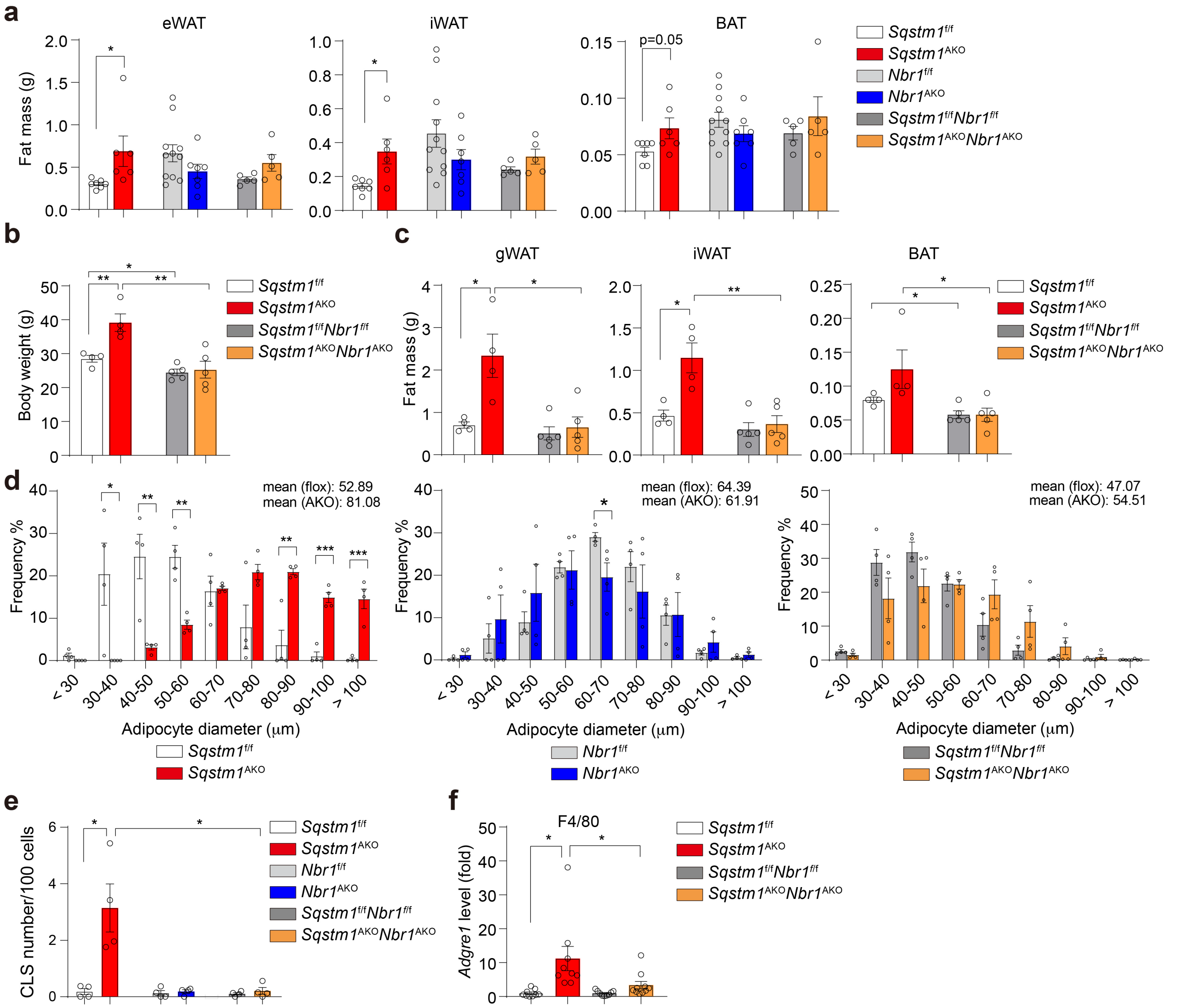

Supplementary Fig. 1. Role of adipocyte *NBR1* in the control of adiposity. a Fat tissue weight of eWAT, iWAT, BAT from adipocyte-specific knock out male mice at 10 - 12 weeks of age. *Sqstm1*$^{f/f}$ (n = 7), *Sqstm1*^{AKO} (n = 6), *Nbr1*$^{f/f}$ (n = 11), *Nbr1*^{AKO} (n = 7), *Sqstm1*$^{f/f}$ *Nbr1*$^{f/f}$ (n = 5) and *Sqstm1*^{AKO}*Nbr1*^{AKO} (n = 5). $p = 0.0417$ (eWAT), $p = 0.0138$ (iWAT) *Sqstm1*^{AKO} vs *Sqstm1*$^{f/f}$. **b**, **c** Body weight (**b**) and fat tissue weight (**c**) of gWAT, iWAT, BAT from adipocyte-specific knock out female mice at 20 - 25 weeks of age. Results are presented as change fold related to individual controls. *Sqstm1*$^{f/f}$ (n = 4), *Sqstm1*^{AKO} (n = 4), *Sqstm1*$^{f/f}$*Nbr1*$^{f/f}$ (n = 5) and *Sqstm1*^{AKO}*Nbr1*^{AKO} (n = 5). gWAT: gonadal WAT. **d** Adipocyte size measurement from H&E staining of eWAT from adipocyte-specific knock out male mice at 25 - 28 weeks of age (n = 4, per genotype). Distribution arrange and frequency was shown. **e** Quantification of CLS number in eWAT from staining described in (**d**) (n = 4, per genotype). CLS: clown-like structure. **f** qPCR analysis of macrophage marker F4/80 (encoded by *Adgre1*) in eWAT. Results are presented as change fold related to individual controls. WT (n = 9), *Sqstm1*$^{-/-}$ (n = 9), *Nbr1*$^{-/-}$ (n = 9) and *Sqstm1*$^{-/-}$*Nbr1*$^{-/-}$ (n = 10). Data are presented as mean ± s.e.m (**a** - **f**). *$p < 0.05$, **$p < 0.01$, ***$p < 0.001$. Two tailed Student's T-test (**a** - **f**). Source data are provided as a Source Data file.

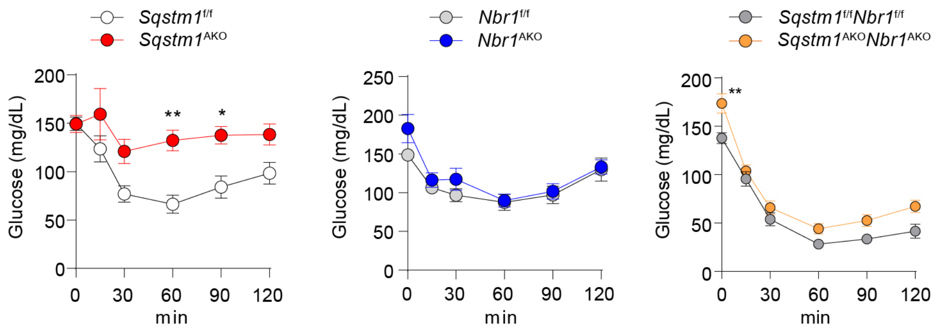

Supplementary Fig. 2. Role of adipocyte *NBR1* in the control of insulin resistance. Insulin tolerance test (ITT) were performed in male mice at 25 - 28 weeks of age. Results are presented as raw values. *Sqstm1*$^{f/f}$ (n = 7), *Sqstm1*^{Ako} (n = 6), *Nbr1*$^{f/f}$ (n = 7), *Nbr1*^{AKO} (n = 6), *Sqstm1*$^{f/f}$*Nbr1*$^{f/f}$ (n = 8) and *Sqstm1*^{AKO}*Nbr1*^{AKO} (n = 11). Data are presented as mean ± s.e.m. $*p < 0.05$, $**p < 0.01$. Two-way ANOVA followed by Bonferroni's post-test. Source data are provided as a Source Data file.

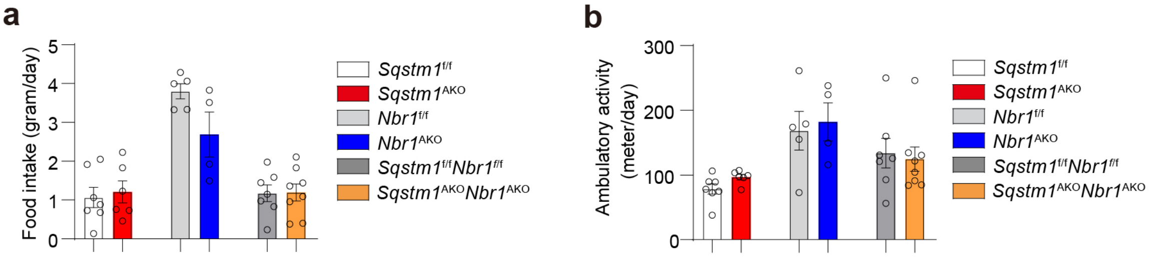

Supplementary Fig. 3. Role of *NBR1* in systemic energy expenditure. a, **b** Food intake (**a**) and sum of all directed ambulatory locomotor activity (**b**) were determined in male mice at 50 - 55 weeks-old using an automated indirect calorimetry system (CLAMS). *Sqstm1*$^{f/f}$ (n = 7), *Sqstm1*^{AKO} (n = 6), *Nbr1*$^{f/f}$ (n = 5), *Nbr1*^{AKO} (n = 4), *Sqstm1*$^{f/f}$*Nbr1*$^{f/f}$ (n = 7) and *Sqstm1*^{AKO}*Nbr1*^{AKO} (n = 8). Results are presented as mean ± s.e.m (**a**, **b**). Two-tailed Student's T-test (**a**, **b**). Source data are provided as a Source Data file.

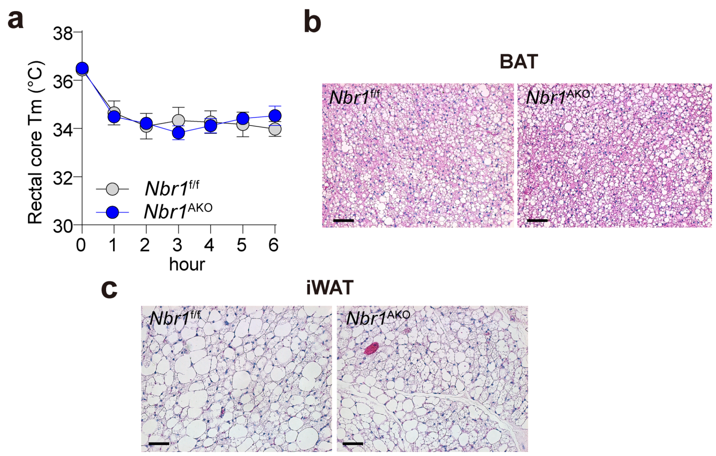

Supplementary Fig. 4. Role of NBR1 in adaptive thermogenesis in BAT and inguinal WAT. **a**,**b** Male mice at 25-weeks of age were subjected to acute cold exposure (4°C) for 7 hours to stimulate brown thermogenesis. **a** Rectal core temperature was measured for consecutive 6 hours. *Nbr1*$^{f/f}$ (n = 6), *Nbr1*^{AKO} (n = 6). Two-way ANOVA followed by Bonferroni's post-test. Data are presented as mean ± s.e.m. **b** Representative H&E staining in BAT of indicated mice (n = 3, per genotype). Scale bar = 100 μm. **c** Male mice at 25-weeks of age were injected with CL316,243 or saline as control for consecutive 5 days. Representative H&E staining in iWAT of indicated mice (n = 3, per genotype). Scale bar = 100 μm. Source data are provided as a Source Data file.

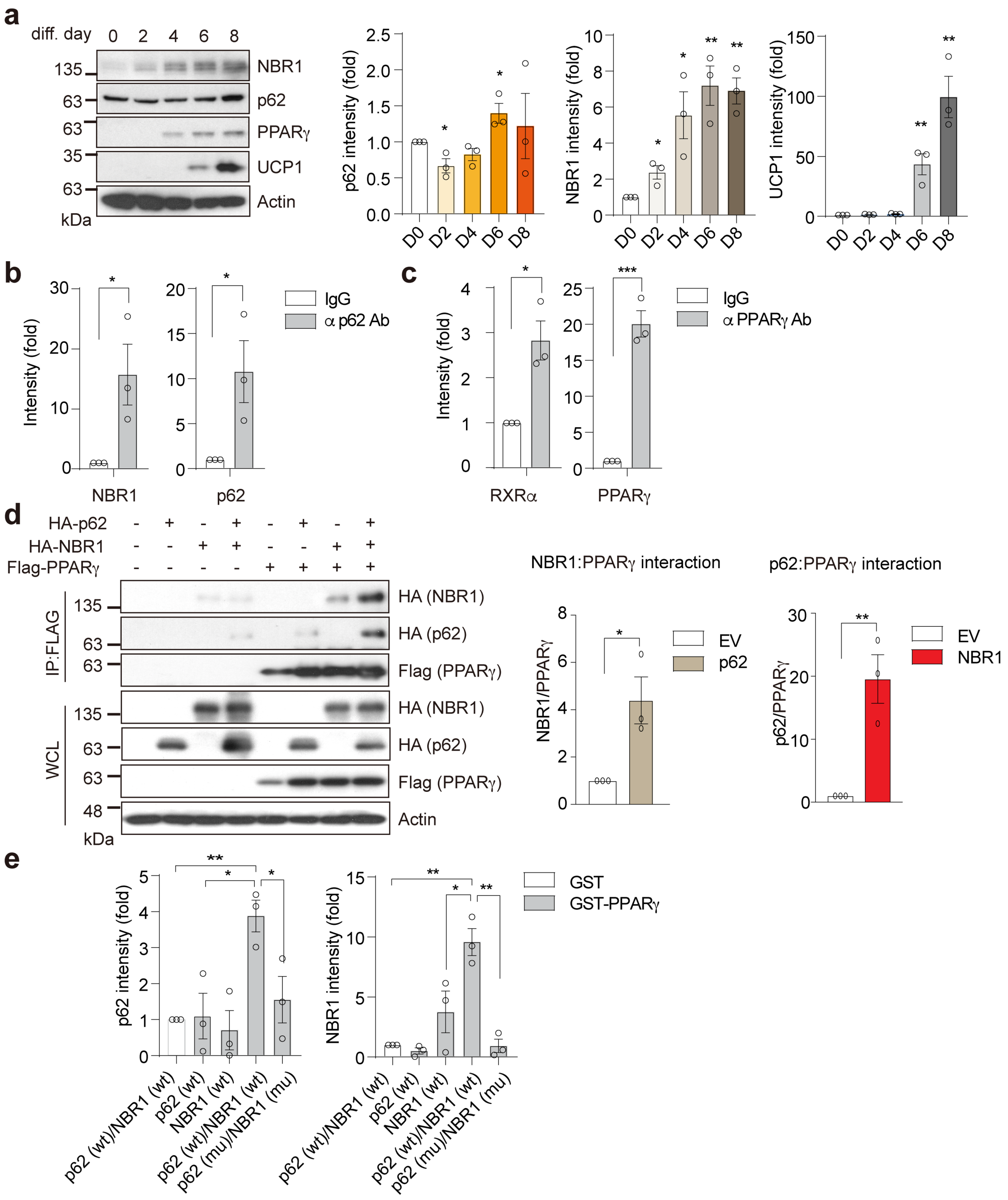

Supplementary Fig. 5. p62 and NBR1 interact with PPARγ and controls its regulation thermogenesis in brown adipocytes. a Representative immunoblotting of p62 and NBR1 levels during brown adipocyte differentiation. Densitometric quantification from 3 independent experiments was shown. **b**, **c** Endogenous interaction of PPARγ with p62 and NBR1. p62 (**b**) or

PPARγ (**c**) immunoprecipitates from nuclear lysates extracted from ISO and rosiglitazone-treated brown adipocytes were analyzed for the levels of specified proteins. Densitometric quantification was shown (n = 3 independent experiments for both). **d** HEK293T cells were transfected with HA-p62, HA-NBR1 and FLAG-tagged PPARγ. Anti-FLAG immunoprecipitates were analyzed by immunoblotting and densitometric quantification was shown (n = 3 independent experiments). **e** HEK293T cells were transfected with cDNA vectors expressing WT/mutants of HA-p62 or HA-NBR1, and GST-PPARγ. The interacting proteins were pulled down using glutathione-beads against GST-PPARγ and analyzed by immunoblotting. Densitometric quantification was shown (n = 3, independent experiments). Data are presented as mean ± s.e.m (**a** - **e**). $^*p < 0.05$, $^{**}p < 0.01$, $^{***}p < 0.001$ for Two tailed Student's T-test (**a** - **e**). Source data are provided as a Source Data file.

Supplementary Table 1. Quantitative PCR primer sequences

**Gene Symbol**	**Forward**	**Reverse**
Ldlr	CAACAATGGTGGCTGTTCCCACAT	ACTCACACTTGTAGCTGCCTTCCA
Fasn	CTTCAACCTGGCCATGGTTTT	GTTGGCGAAGCCGTAGTTAGTT
Srebf1	TATGGAGGGCATGAAACCCGAAGT	TTGACCTGGCTATCCTCAAAGGCT
Srebf2	ATGGAGACCCTCACGGA	TGCTGTTGTTGCCACTG
Hmgcr	TCAGTGGGAACTATTGCACCG	TGGAATGACGGCTTCACAAAC
Ucp1	TCTTCTCAGCCGGAGTTTCAGCTT	ACCTTGGATCTGAAGGCGGACTTT
Dio2	AAGGCTGCCGAATGTCAACGAATG	TGCTGGTTCAGACTCACCTTGGAA
Cox7α	CAGCGTCATGGTCAGTCTGT	AGAAAACCGTGTGGCAGAGA
Pgc1a	AGCTGTGTTTGACGACAAATC	CGACACGGAGAGTTAAAGGAAG
CIDEα	ATCACAACTGGCCTGGTTACG	TACTACCCGGTGTCCATTTCT
Adgre1	TGTCTGACAATTGGGATCTGCCCT	ATAGCTTCCGAGAGTGTTGTGGCA
18s	GTAACCCGTTGAACCCATT	CCATCCAATCGGTAGTAGCG
