# Supplementary material for: NBR1 is a critical step in the repression of thermogenesis of p62-deficient adipocytes through PPARγ
Source: Nat Commun. 2021 May 17;12:2876. doi: 10.1038/s41467-021-23085-0 (PMC8129077; doi:10.1038/s41467-021-23085-0)
Supplement: Supplementary file 2 — Reporting Summary [file 41467_2021_23085_MOESM2_ESM.pdf]

nature research

Corresponding author(s): Jorge Moscat

Last updated by author(s): Apr 1, 2021

Reporting Summary

Nature Research wishes to improve the reproducibility of the work that we publish. This form provides structure for consistency and transparency in reporting. For further information on Nature Research policies, see Authors & Referees and the Editorial Policy Checklist .

Statistics

For all statistical analyses, confirm that the following items are present in the figure legend, table legend, main text, or Methods section.

n/a	Confirmed	
☐	☒	The exact sample size (*n*) for each experimental group/condition, given as a discrete number and unit of measurement
☐	☒	A statement on whether measurements were taken from distinct samples or whether the same sample was measured repeatedly
☐	☒	The statistical test(s) used AND whether they are one- or two-sided<br>*Only common tests should be described solely by name; describe more complex techniques in the Methods section.*
☒	☐	A description of all covariates tested
☐	☒	A description of any assumptions or corrections, such as tests of normality and adjustment for multiple comparisons
☐	☒	A full description of the statistical parameters including central tendency (e.g. means) or other basic estimates (e.g. regression coefficient) AND variation (e.g. standard deviation) or associated estimates of uncertainty (e.g. confidence intervals)
☐	☒	For null hypothesis testing, the test statistic (e.g. *F*, *t*, *r*) with confidence intervals, effect sizes, degrees of freedom and *P* value noted<br>*Give P values as exact values whenever suitable.*
☒	☐	For Bayesian analysis, information on the choice of priors and Markov chain Monte Carlo settings
☒	☐	For hierarchical and complex designs, identification of the appropriate level for tests and full reporting of outcomes
☒	☐	Estimates of effect sizes (e.g. Cohen's *d*, Pearson's *r*), indicating how they were calculated

Our web collection on statistics for biologists contains articles on many of the points above.

Software and code

Policy information about availability of computer code

Data collection	Bio-Rad CFX Maestro 1.1 (version 4.1.2433.1219) is used to collect qRT-PCR data using the Bio-Rad CFX96 Touch™ Real-Time PCR Detection System.<br>AxioVision LE (version 4.8.2.0) was used to capture microscopy image.<br>Agilent Seahorse Wave desktop software (version 2.6.1) was used to acquire seahorse data.
Data analysis	ImageJ (version 1.50b.) (https://imagej.nih.gov/ij/) was used for densitometric quantification of immunoblot images.<br>Adiposoft (version 1.16.) (https://imagej.net/Adiposoft) was used to quantify the adipocyte size and number.<br>Graphpad Prism (Version 8.3.0) (https://www.graphpad.com/scientific-software/prism/) was used for statistical analyses and graph visualization.<br>SPSS Statistics (version 24) (https://www.ibm.com/products/spss-statistics) was used for ANCOVA statistical analysis.

For manuscripts utilizing custom algorithms or software that are central to the research but not yet described in published literature, software must be made available to editors/reviewers. We strongly encourage code deposition in a community repository (e.g. GitHub). See the Nature Research guidelines for submitting code & software for further information.

Data

Policy information about availability of data

All manuscripts must include a data availability statement. This statement should provide the following information, where applicable:

- Accession codes, unique identifiers, or web links for publicly available datasets
- A list of figures that have associated raw data
- A description of any restrictions on data availability

The data that support the findings of this study are provided in the data source file and available from the corresponding author upon reasonable request. The source data underlying Figs. 1a-d, f-i, 2a-e, g, h, 3a-f, 4a-d, 5a, c, e-g, 6a-d, 7a-f, 8a-e, Supplementary Figs. 1a-f, 2, 3a, 3b, 4a, 5a-e are provided as a Source data file. Also, the statistical p-value from GraphPad Prizm or SPSS reports is provided the individual figure legends. Notably, in case of significance with p<0.0001, GraphPad

Prizm V.8.3.0 does not give exact p-values and rather states that significance is p<0.0001.

Field-specific reporting

Please select the one below that is the best fit for your research. If you are not sure, read the appropriate sections before making your selection.

- [x] Life sciences
- [ ] Behavioural & social sciences
- [ ] Ecological, evolutionary & environmental sciences

For a reference copy of the document with all sections, see nature.com/documents/nr-reporting-summary-flat.pdf

Life sciences study design

All studies must disclose on these points even when the disclosure is negative.

Sample size

We did not perform statistical calculations of sample-size. Sample size was determined based on reproducibility between biological replicates and independent experiments as well as the magnitude and consistency of measurable differences between groups. For animal experiments, cohort size was determined by types of experiment and availability of animals as littermates were used in each experiment.

Data exclusions

We have not excluded any data.

Replication

Animal experiments were independently performed for at least two times with similar findings. In vitro data have been performed independently for at least three times with similar results. Despite technical problems (eg: poor protein or RNA quality after cell/tissue lysis, low primary cell viability after isolation, poor cell differentiation rate, poor DNA transfection efficiency or pipetting errors) all replicates show similar results.

Randomization

Subject animals using global knockout mice were randomly assigned in animal experiments but the animals from the tissue-specific knockout lines were not randomly assigned because flox and cre genotyping of each subject to discriminate the wild-type and knockout is required prior to the animal experiments. Sex- and age-matched animals were allocated from each genotype into experimental groups. For in vitro cell based experiments, individual wells were randomized into treatment groups and were processed at the same time to control for potential covariates.

Blinding

For animal experiment, investigators were not blinded to genotypes and treatment conditions because information on sex, age and genotypes is prerequisite for the group assignment.

Reporting for specific materials, systems and methods

We require information from authors about some types of materials, experimental systems and methods used in many studies. Here, indicate whether each material, system or method listed is relevant to your study. If you are not sure if a list item applies to your research, read the appropriate section before selecting a response.

Materials & experimental systems

n/a	Involved in the study	
☐	☒	Antibodies
☐	☒	Eukaryotic cell lines
☒	☐	Palaeontology
☐	☒	Animals and other organisms
☒	☐	Human research participants
☒	☐	Clinical data

Methods

n/a	Involved in the study	
☒	☐	ChIP-seq
☒	☐	Flow cytometry
☒	☐	MRI-based neuroimaging

Antibodies

Antibodies used

1. mouse monoclonal anti-GAPDH antibody (Santa Cruz Biotech; #sc-32233; clone:6C5; RRID:AB_627679; Dilution: 1:20,000),
2. mouse monoclonal anti-β-actin antibody (Sigma-Aldrich; #A1978; clone:AC-15; RRID:AB_476692; Dilution 1:20,000),
3. Rabbit polyclonal anti-Histone H3 antibody (Abcam; #ab1791; RRID:AB_302613; Dilution 1:25,000),
4. Rabbit polyclonal anti-UCP1 antibody (Abcam; #ab10983; RRID:AB_2241462; Dilution: 1:6,000),
5. mouse monoclonal anti-alpha Tubulin antibody (Santa Cruz Biotech; #sc-8035; clone:TU-02; RRID:AB_628408; Dilution: 1:1000),
6. rabbit monoclonal anti-phospho-CREB (Ser133) antibody (Cell signaling; #9198; clone 87G3; RRID:AB_2561044; Dilution 1:1,000),
7. mouse monoclonal anti-PPARgamma antibody (Santa Cruz Biotech; #sc-7273; clone:E-8; RRID:AB_628115; Dilution 1:800),
8. rabbit monoclonal anti-PPARgamma antibody (Cell signaling; #2443; clone:81B8; Dilution 1:1,000),
9. rabbit polyclonal anti-RXRα antibody (Santa Cruz Biotech; #sc-553; clone:D-20; RRID:AB_2184874; Dilution 1:500),
10. rabbit polyclonal anti-RXRα/β/γ antibody (Santa Cruz Biotech; #sc-774; RRID:AB_2270041; Dilution 1:500),
11. rabbit monoclonal anti-p62 antibody (Rodent Specific) (Cell signaling; #23214; clone:D6M5X; RRID:AB_2798858; Dilution

1:1000),
12. guinea pig polyclonal anti-p62 antibody (Progen; #GP62-C; RRID:AB_2687531; Dilution 1:1000),
13. rabbit polyclonal anti-p62 antibody (Thermo Scientific; #PA5-20839; RRID:AB_11157045; Dilution 1:1000),
14. mouse monoclonal anti-NBR1 antibody (Santa Cruz Biotech; #sc-130380; clone:4BR; RRID:AB_2149402; Dilution 1:800),
15. mouse monoclonal anti-SDHB antibody (Santa Cruz Biotech; #sc-271548; clone:G-10; RRID:AB_10659104; Dilution 1:2,000),
16. mouse monoclonal anti-UQCRC2 antibody (Santa Cruz Biotech; #sc-390378; clone:G-10; RRID:AB_2754980; Dilution 1:2,000),
17. mouse monoclonal anti-GST antibody (Santa Cruz Biotech; #sc-138; clone:B-14; RRID:AB_627677; Dilution 1:500),
18. mouse monoclonal anti-HA antibody (Santa Cruz Biotech; #sc-7392; clone:F-7; RRID:AB_627809; Dilution 1:500),
19. mouse monoclonal anti-FLAG antibody (Sigma-Aldrich; #F1804; clone:M2; RRID:AB_262044; Dilution 1:4,000),
20. mouse monoclonal anti-Myc antibody (Santa Cruz Biotech; #sc-40; clone:9E10; RRID:AB_2857941; Dilution 1:500),
21. rat monoclonal anti-mouse IgG1 antibody (BD Biosciences; #550331; clone:A85-1; RRID:AB_2296342; Dilution 1:3,000),
22. goat polyclonal anti-rabbit IgG antibody (Dako; #E0432; RRID:AB_2313609; Dilution 1:3,000)

Validation

1. https://datasheets.scbt.com/sc-32233.pdf
2. https://www.sigmaaldrich.com/catalog/product/SIGMA/A1978?lang=en&region=US
3. https://www.abcam.com/histone-h3-antibody-nuclear-marker-and-chip-grade-ab1791.html
4. https://www.abcam.com/ucp1-antibody-ab10983.html
5. https://datasheets.scbt.com/sc-8035.pdf
6. https://www.cellsignal.com/products/primary-antibodies/phospho-creb-ser133-87g3-rabbit-mab/9198
7. https://datasheets.scbt.com/sc-7273.pdf
8. https://www.cellsignal.com/products/primary-antibodies/pparg-81b8-rabbit-mab/2443
9. https://datasheets.scbt.com/sc-553.pdf
10. https://www.citeab.com/antibodies/827411-sc-774-rxr-antibody-n-197
11. https://www.cellsignal.com/products/primary-antibodies/sqstm1-p62-d6m5x-rabbit-mab-rodent-specific/23214
12. https://www.progen.com/anti-p62-sqstm1-c-terminus-guinea-pig-polyclonal-serum.html?___store=us
13. https://www.thermofisher.com/antibody/product/SQSTM1-Antibody-Polyclonal/PA5-20839
14. https://datasheets.scbt.com/sc-130380.pdf
15. https://datasheets.scbt.com/sc-271548.pdf
16. https://datasheets.scbt.com/sc-390378.pdf
17. https://datasheets.scbt.com/sc-138.pdf
18. https://datasheets.scbt.com/sc-7392.pdf
19. https://www.sigmaaldrich.com/catalog/product/sigma/f1804?lang=en&region=US&cm_sp=Insite-_-caSrpResults_srpRecs_srpModel_p2983-_-srpRecs3-2
20. https://datasheets.scbt.com/sc-40.pdf
21. https://www.citeab.com/antibodies/search?q=550331
22. https://www.citeab.com/antibodies/search?q=Dako%3B+%23E0432

Eukaryotic cell lines

Policy information about cell lines

Cell line source(s)

HEK293T, HEK293-FT cell lines were purchased from ATCC. Wild-type immortalized BAT SVF is a gift from Shingo Kajimura at Harvard University. The primary brown adipocytes and immortalized brown adipocytes were generated in house.

Authentication

No authentication was performed for cell lines purchased from ATCC. For immortalized BAT SVF, cells were tested for differentiation capacity and UCP1 expression (brown cell marker) and specific knockout lines were confirmed by assessing protein expression of target genes.

Mycoplasma contamination

Cell lines were tested routinely and were all negative for mycoplasma contamination.

Commonly misidentified lines
(See ICLAC register)

No commonly misidentified cell lines were used.

Animals and other organisms

Policy information about studies involving animals; ARRIVE guidelines recommended for reporting animal research

Laboratory animals

Generation of Sqstm1-/- (Rodriguez A, et al, Cell Metab, 2006), Sqstm1 fl/fl (Muller TD, et al, J Clin Invest, 2013) and Nbr1 fl/fl (Hernandez ED, et al, Cell Metab, 2014) mice were previously described. aP2-Cre mice were purchased from Jackson Laboratory (stock number 005069). All mouse strains were generated in a C57BL/6 background. Mice were fed a normal chow diet and kept on a 12h light/12h dark cycle with free access to food and water in a temperature (22 ± 1°C) and humidity (50 ± 5%) controlled room.

Fig.1: 10-12 week old wild-type or total body knockout male mice (WT, Sqstm1-/-, Nbr1-/- or Sqstm1-/-Nbr1-/-).
Fig.2a: 13-24 week old aP2-cre positive Sqstm1 fl/fl, Nbr1 fl/fl and dual fl/fl or aP2-cre negative fl/fl male mice.
Fig.2b-h, Fig.3, Fig.8b, Supplementary Fig.1d-f, Supplementary Fig.2: 25-28 week old aP2-cre positive Sqstm1 fl/fl, Nbr1 fl/fl and dual fl/fl or aP2-cre negative fl/fl male mice.

	Fig.4, Supplementary Fig.3: 50-55 week old aP2-cre positive Sqstm1 fl/fl, Nbr1 fl/fl and dual fl/fl or aP2-cre negative fl/fl male mice.<br>Fig.5, Supplementary Fig.4: 25 week old aP2-cre positive Sqstm1 fl/fl, Nbr1 fl/fl and dual fl/fl or aP2-cre negative fl/fl male mice.<br>Fig.6a,b, Fig.7a: 0-2 day old male/female neonates for isolation of stromal vascular fractions (SVF).<br>Supplementary Fig.1a 10-12 week old aP2-cre positive Sqstm1 fl/fl, Nbr1 fl/fl and dual fl/fl or aP2-cre negative fl/fl male mice.<br>Supplementary Fig.1b 20-25 week old aP2-cre positive Sqstm1 fl/fl, Nbr1 fl/fl and dual fl/fl or aP2-cre negative fl/fl female mice.
Wild animals	The study did not involve wild animals.
Field-collected samples	The study did not involve samples collected from the field.
Ethics oversight	Animal handling and experimental procedures conformed to institutional guidelines and were approved by the SBP Medical Discovery Institute Institutional Animal Care and Use Committee.

Note that full information on the approval of the study protocol must also be provided in the manuscript.
